# Fuzzy Strategy Grey Wolf Optimizer for Complex Multimodal Optimization Problems

**DOI:** 10.3390/s22176420

**Published:** 2022-08-25

**Authors:** Hua Qin, Tuanxing Meng, Yuyi Cao

**Affiliations:** College of Computer and Electronic Information Engineering, Guangxi University, Nanning 530004, China

**Keywords:** multimodal optimization problems, grey wolf optimizer, fuzzy search direction, fuzzy crossover operator, binary joint normal distribution

## Abstract

Traditional grey wolf optimizers (GWOs) have difficulty balancing convergence and diversity when used for multimodal optimization problems (MMOPs), resulting in low-quality solutions and slow convergence. To address these drawbacks of GWOs, a fuzzy strategy grey wolf optimizer (FSGWO) is proposed in this paper. Binary joint normal distribution is used as a fuzzy method to realize the adaptive adjustment of the control parameters of the FSGWO. Next, the fuzzy mutation operator and the fuzzy crossover operator are designed to generate new individuals based on the fuzzy control parameters. Moreover, a noninferior selection strategy is employed to update the grey wolf population, which makes the entire population available for estimating the location of the optimal solution. Finally, the FSGWO is verified on 30 test functions of IEEE CEC2014 and five engineering application problems. Comparing FSGWO with state-of-the-art competitive algorithms, the results show that FSGWO is superior. Specifically, for the 50D test functions of CEC2014, the average calculation accuracy of FSGWO is 33.63%, 46.45%, 62.94%, 64.99%, and 59.82% higher than those of the equilibrium optimizer algorithm, modified particle swarm optimization, original GWO, hybrid particle swarm optimization and GWO, and selective opposition-based GWO, respectively. For the 30D and 50D test functions of CEC2014, the results of the Wilcoxon signed-rank test show that FSGWO is better than the competitive algorithms.

## 1. Introduction

Many complex optimization problems in industrial applications have multiple global optimal solutions or near-optimal solutions that provide decision-makers with different decision preferences, and such problems are often referred to as multimodal optimization problems (MMOPs) [1]. Air service network design is an example of a MMOP, which requires all feasible routes to transport goods to the destination; if the current route cannot be executed due to weather conditions, an alternative route with a similar cost can be selected from the feasible routes to transport goods [2]. Other examples of MMOPs include structural damage detection [3], image segmentation [4], flight control systems [5], job shop scheduling [6], truss structure optimization [7], protein structure prediction [8], and electromagnetic design [9]. Most MMOPs are nonconvex and nonlinear; classical numerical optimization methods are sensitive to nonconvexity and nonlinearity, so they encounter difficulties in solving MMOPs. In contrast, evolutionary algorithms are not sensitive to the nonconvexity and nonlinearity of optimization problems and have been widely used to solve MMOPs, such as genetic algorithms [10], evolutionary algorithms [11], particle swarm optimization [12], ant colony optimization [13], cuckoo search algorithms [14], memetic algorithms [15], niching chaos optimization [16], grey wolf optimization [17], harmony search algorithms [18], fireworks algorithms [19], and gravitational search algorithms [20]. Among these evolutionary algorithms for solving MMOPs, grey wolf optimizers (GWOs) have the advantages of easy implementation and requiring few parameters [21]. Moreover, GWOs use three leading wolves to guide the search and more easily escape local optima, making GWOs competitive for solving MMOPs [22,23,24]. Because the solution space of MMOPs is very complex, GWOs have difficulty balancing convergence and diversity [25,26], so GWOs cannot easily estimate the position of the optimal solution, and the obtained solutions are relatively poor [27]. To address these drawbacks of GWOs, many improved variants of GWOs have been developed, which can be divided into two categories. The first category is to improve the control parameters of GWOs. GWOs have two control parameters, *a* and *C*; the former is a linearly attenuated search step, and the latter is the neighborhood radius coefficient, both of which can be used to control the diversity and convergence of GWOs. In [28,29,30,31], methods such as nonlinear functions and chaotic sequences are proposed to construct parameter *a*, which further enhances the diversity of individuals and improves the ability of GWOs to bounce from local optima. In most GWOs, *a* and *C* are treated as independent parameters, while the opinion presented in [32] is that they are related, and a method to calculate *C* with *a* is proposed, which further enhances the diversity of GWOs. The second category is to design new individual update strategies. Individual update strategies, such as Levy flight [33,34], Cauchy operator [35], opposition-based learning [36], refraction learning [37], and chaotic opposition-based approaches [38], can make some individuals in the population change greatly, thereby increasing the diversity of GWOs. Update strategies of other evolutionary algorithms, such as the whale optimization algorithm (WOA) [39], covariance matrix adaptation-evolution strategy (CMA-ES) [40], minimum conflict algorithm (MCA) [41], and grasshopper optimization algorithm (GOA) [42], are applied to improve the individual update strategies of GWOs and can also effectively improve the diversity and convergence of GWOs.

By using GWOs to solve MMOPs, the convergence speed and quality of solutions must be further improved, and there are three main reasons for these defects. (i) The absolute value operation in the search direction leads to the loss of negative signs in some dimensions, resulting in an incorrect search and affecting the speed of convergence. (ii) The new individuals of GWOs are mutated in all dimensions, which is a phenomenon of search divergence and affects the convergence and quality of solutions. (iii) GWOs allow worse new individuals to be updated into the population, resulting in the population being unable to effectively estimate the region where the optimal solution is located and reducing the convergence speed of the algorithm. To address these drawbacks of GWOs, a fuzzy strategy grey wolf optimizer (FSGWO) for MMOPs is proposed in this paper. The role of the fuzzy strategy is to automatically adjust the control parameters of the algorithm to realize the adaptive balance of diversity and convergence. By using fuzzy control parameters, new evolutionary operations are designed to increase the algorithm’s abilities to explore new regions and local search, and to improve the quality of the solutions to MMOPs. The main contributions of this paper are as follows: (i) A new grey wolf individual update strategy is proposed. First, both global and local search information is added to the fuzzy search direction, which is used to guide individual mutation and enhance the individual’s ability to detect the optimal solution. Second, the fuzzy cross-operator is applied to generate new individuals and avoid the mutation of new individuals in all dimensions, which is a method to control the phenomenon of individual search divergence. Finally, a noninferior selection strategy is employed to update the population, allowing only better new individuals to be updated into the population, which improves the ability of the grey wolf population to estimate the location of the optimal solution and helps accelerate the convergence. (ii) Binary joint normal distribution is used as a fuzzy method to realize the adaptive adjustment of the control parameters of FSGWO. The two control parameters of the FSGWO are considered to have an intrinsic correlation, which is modeled by a binary joint normal distribution. In the iterative process, the parameters of the binary joint normal distribution method are adaptively updated with information about the current optimal solutions to automatically control the convergence speed. (iii) The FSGWO is verified on the 30 30D and 50D test functions of IEEE CEC2014 and five engineering application problems and compared with state-of-the-art competitive algorithms. The results show that the proposed algorithm has advantages over competitive algorithms in solving MMOPs, and the proposed improvement ideas are feasible for balancing the diversity and convergence of traditional GWOs. Specifically, for the 50D test functions of CEC2014, the average calculation accuracy of FSGWO is 33.63%, 46.45%, 62.94%, 64.99%, and 59.82% higher than those of the equilibrium optimizer algorithm (EO), modified particle swarm optimization (MPSO), original GWO, hybrid particle swarm optimization and grey wolf optimizer (HPSOGWO), and selective opposition-based GWO (SOGWO), respectively, which indicates that the FSGWO can significantly improve the calculation accuracy when solving high-dimensional MMOPs. For the 30D and 50D test functions of CEC2014, the results of the Wilcoxon signed-rank test show that the proposed algorithm is better than the competitive algorithms.

The remainder of this paper is arranged as follows: Section 2 introduces the principle of the original GWO. Section 3 provides the key details and the flowchart of FSGWO. Section 4 presents the experimental results, and Section 5 is the discussion. Finally, Section 6 summarizes the study.

## 2. Related Work

### 2.1. Algorithm Flow of the Original Grey Wolf Optimizer

The grey wolf population is denoted by *X* = {$X_{1}$, $X_{2}$, *…*, *$X_{M}$*}, where *M* is the number of grey wolves. *X_p_* is an individual in *X*. In the original GWO [21], the three best solutions appearing in the iterative process are called the three leading wolves and are denoted by $X_{\alpha }$, $X_{\beta }$, and $X_{\delta }$. The three leading wolves are used to guide grey wolf individuals to round up prey, which is also known as updating individuals.

The search direction $D_{\alpha }$ from $X_{p}$ to $X_{\alpha }$ is calculated as

(1) $$D_{\alpha }\;=\;\left|C_{1}\;⊙\;X_{\alpha }\;-\;X_{p}\right|$$

where $C_{1}$ is the neighborhood radius, which is a random vector between (0, 2). The operator ⨀ represents the vector dot product operation. *C*_1_ ⨀ $X_{\alpha }$ is a neighborhood point of $X_{\alpha }$. The operator |∙| is an absolute value operator.

A mutant individual of $X_{p}$ generated by $X_{\alpha }$ is written as

(2) $$X_{1}\;=\;X_{\alpha }\;-\;A_{1}\;⊙\;D_{\alpha }$$

where $A_{1}$ is the search step, which is a random vector between (−2, 2).

Similarly, a mutant individual of $X_{p}$ generated by $X_{\beta }$ can be presented as

(3) $$X_{2}\;=\;X_{\beta }\;-\;A_{2}\;⊙\;D_{\beta }$$

where *D_β_* = |$C_{2}$ ⨀ $X_{\beta }$ − $X_{p}$| is the search direction. *C*_2_ is a random vector between (0, 2), and $A_{2}$ is a random vector between (−2, 2).

In the same way, a mutant individual of *X_p_* generated by $X_{\delta }$ is

(4) $$X_{3}\;=\;X_{\delta }\;-\;A_{3}\;⊙\;D_{\delta }$$

where *D_δ_* = |$C_{3}$ ⨀ *X_δ_* − $X_{p}$| is the search direction. *C*_3_ is a random vector between (0, 2), and $A_{3}$ is a random vector between (−2, 2).

The new individual *X_u_*, generated from the above three mutant individuals, can be expressed as

(5) $$X_{u}\;=\;\frac{X_{1}\;+\;X_{2}\;+\;X_{3}}{3}$$

Finally, *X_p_* in *X* is replaced with *X_u_*, completing the update of the individual $X_{p}$.

This update strategy of GWO has two drawbacks. (i) Compared with *X_p_*, all dimensions of *X_u_* are mutated, which leads to divergence when solving high-dimensional MMOPs and reduces the quality of solutions. (ii) Moreover, GWO uses only three leading wolves as heuristic information to guide individuals to search for the optimal solution. The distribution of the optimal solutions of MMOPs is more complex, and the three leading wolves cannot quickly estimate the region of the optimal solution; thus, the algorithm slowly converges.

The pseudocode of the original GWO algorithm is shown in Algorithm 1.
**Algorithm 1.** Pseudocode of the original GWO algorithm [21].Initialize the grey wolf population *X_i_* (*i* = 1, 2, …, *M*)Initialize *a*, *A*, and *C*Calculate the fitness of each search agent$X_{\alpha }$ = the best search agent*X_β_* = the second-best search agent$X_{\delta }$ = the third-best search agentwhile (*t* < Max number of iterations)   for each search agent    Update the position of *X_i_*   end for   Update *a*, *A*, and *C*   Calculate the fitness of all search agents   Update *X_α_*, $X_{\beta }$, and *X_δ_*   *t* = *t* + 1end whilereturn $X_{\alpha }$

### 2.2. Fuzzy Adaptive Control Parameters

For GWOs, the design of adaptive control parameters is a challenge. In [28,29,30,31], nonlinear functions are used to design adaptive control parameters for GWOs, but such adaptive parameters are not effective in solving MMOPs, and the quality of the obtained solutions is not high. The main reason for this result is that the nonlinear function cannot know the complexity of the solution space of MMOPs and cannot use the current iteration information to estimate the position of the optimal solution.

In recent studies, fuzzy methods have been used to address the issues of adaptive control parameters of evolutionary algorithms. To achieve an optimal balance of exploitation and exploration in the chicken swarm optimization algorithm [43], the fuzzy system is applied to adaptively adjust the number of chickens and random factors. In [44], the fuzzy system is used for the design of the crossover rate control parameter of the differential evolution algorithm, which improves the diversity of the population. In [45], the fuzzy inference system is employed to automatically tune the control parameters of the whale optimization algorithm, which improves the convergence of the algorithm. The common feature of these fuzzy methods is updating the control parameters with the information of the optimal solution in the current iteration.

Fuzzy methods provide new ideas for the design of adaptive control parameters for GWOs. Inspired by this, in this study, bivariate joint normal distribution is used as a fuzzy method to design adaptive control parameters of GWO and new evolutionary operators are designed based on these fuzzy control parameters. Finally, the improved GWO is employed to solve MMOPs.

## 3. The Proposed Algorithm

**Figure 1:**
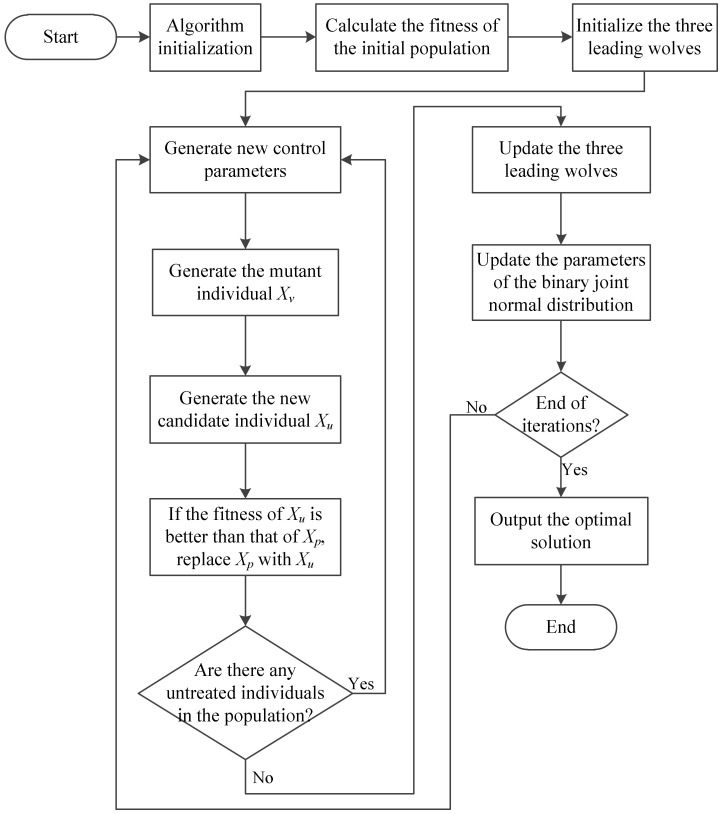
Flowchart of FSGWO.

The flowchart of the FSGWO is shown in Figure 1. The algorithm parameters and population are initialized first; the fitness of each individual in the initial population is calculated, and the three individuals with the best fitness values are selected as the three initial leading wolves. In the iterative part of the algorithm, new control parameters are obtained by sampling the binary joint normal distribution, and then a new individual *X_u_* is generated through mutation and crossover operations. If *X_u_* is better than *X_p_*, $X_{p}$ is replaced with *X_u_*; otherwise, it is not replaced. Finally, the parameters of the bivariate joint normal distribution are adaptively updated and the algorithm continues to the next iteration. After the end of the iterations, the best leader wolf is taken as the optimal solution and output.

The key points of the FSGWO are described below.

### 3.1. Mutation Strategy with a Fuzzy Search Direction

The mutation of the grey wolf is realized by adding a fuzzy search direction to the grey wolf. The term fuzzy search direction refers to the product of the fuzzy step *r_a_* and the search direction $D_{c}$, and its calculation method is described as follows.

First, three leading wolves are used to estimate the current position *X_c_* of the prey, and $X_{c}$ is given by

(6) $$X_{c}\;=\;\frac{X_{\alpha }\;+\;X_{\beta }\;+\;X_{\delta }}{3}$$

The fuzzy search direction of *X_p_* is defined as

(7) $$D_{c}\;=\;\left(X_{c}\;-\;X_{p}\right)\;+\;\left(X_{p1}\;-\;X_{p2}\right)$$

where $X_{p1}$ and $X_{p2}$ are two individuals randomly selected in *X*, and $X_{p}$ *≠* $X_{p1}$ *≠* $X_{p2}$. The expression *X_c_ −* $X_{p}$ represents the search information from *X_p_* to the prey, which belongs to the global search information. The expression $X_{p1}$ *−* $X_{p2}$ represents the search information between individuals and belongs to the local search information.

There are three differences between Equation (7) and Equation (1). (i) Equation (7) has no absolute value operator and retains the heuristic effect of negative signs on the search. (ii) In the early stage of iterations, the positions of the three leading wolves are generally scattered. Therefore, Equation (7) uses the average value of the three leading wolves to estimate the position of the prey, which can reduce the adverse effects caused by the dispersion of guiding positions and help accelerate the convergence. (iii) Grey wolves have the habit of hunting collectively and surround the prey by exchanging information on the location of their prey. Equation (7) uses $X_{p1}$ − $X_{p2}$ to realize the exchange of prey location information between grey wolves, but there is no method for exchanging prey location information between grey wolves in Equation (1).

The mutant individual $X_{\nu }$ of *X_p_* is generated by the fuzzy search direction, written as

(8) $$X_{\nu }\;=\;X_{p}\;+\;r_{a}\;⊙\;D_{c}$$

where $r_{a}$ is the fuzzy step, which is a random vector between (0, 1). *r_a_* is a control parameter of FSGWO, and its generation method is described later. The expression $r_{a}$ ⨀ *D_c_* represents the fuzzy search direction, which is the product of the fuzzy step $r_{a}$ and the search direction *D_c_*. The expression *X_p_* + $r_{a}$ ⨀ *D_c_* starts from $X_{p}$ and searches for prey in the fuzzy search direction of *r_a_* ⨀ $D_{c}$.

### 3.2. Fuzzy Crossover Operator

All dimensions of *X_ν_* are mutated. To make the search stable, selecting the values on some dimensions from $X_{\nu }$ and then copying them into the corresponding dimensions of *X_u_* is necessary. This is achieved by a fuzzy crossover operator. The term fuzzy crossover operator refers to a crossover operator that uses the fuzzy crossover factor *r_b_*.

The term *j* is denoted as the *j*th dimension of $X_{p}$ and *X_ν_*. The crossover operation on the *j*th dimension can be expressed by

(9) $$X_{u}^{j}\;=\;\left\{\begin{array}{ll} X_{v}^{j}, & \;\mathrm{if}\;r\;\geq \;r_{b}^{j}\\ X_{p}^{j}, & \;\mathrm{else} \end{array}\right.$$

where $r_{b}$ is the fuzzy crossover factor, which is a random vector between (0, 1). $r_{b}^{j}$ is the value in the *j*th dimension of $r_{b}$. *r* is a random number (scalar) that follows the standard uniform distribution. The expression *r* ≥ $r_{b}^{j}$ indicates that the value of the *j*th dimension of $X_{\nu }$ is copied to the *j*th dimension of *X_u_* using the roulette strategy.

After completing the operation of Equation (9), a dimension *w* is randomly specified, and then the mutation operation $X_{u}^{\omega }$ = $X_{y}^{\omega }$ is performed to generate a new individual *X_u_*.

The term fuzzy control parameter means that control parameters $r_{a}$ and *r_b_* are not directly related in formulas, but they can affect the diversity and the convergence of FSGWO, and there is an inherent fuzzy correlation between $r_{a}$ and *r_b_*. Therefore, these two control parameters are related in this paper, and a bivariate joint normal distribution is used to describe that fuzzy relationship. The expression $r_{c}^{j}$ = [$r_{a}^{j}$, $r_{b}^{j}$] is a binary variable, where $r_{a}^{j}$ and $r_{b}^{j}$ are values on the *j*th dimension of $r_{a}$ and *r_b_*, respectively. $r_{c}^{j}$ follows a binary joint normal distribution with a mean of *μ* and a covariance of *∑*, denoted as

(10) $$r_{c}^{j}\;∼\;N(\mu ,\Sigma )$$

where *u* = [$u_{ra}$, $u_{rb}$], *u*_ra_ and $u_{rb}$ are both scalars. The covariance matrix *∑* is defined as

(11) $$\Sigma \;=\;\left[\begin{array}{cc} s_{1}\;\times \;s_{2} & 0\\ 0 & s_{1}\;\times \;s_{3} \end{array}\right]$$

where *s*_1_ is a random number (scalar) following the standard uniform distribution. The terms $s_{2}$ and *s*_3_ are random numbers (scalars) following the standard normal distribution. The values of $s_{2}$ and *s*_3_ obtained by sampling the standard normal distribution may be greater than 1, so the diagonal elements of ∑ have diversity, which makes $r_{c}^{j}$ also have diversity.

By sampling Equation (10), a matrix $r_{c}$ with *d* rows and two columns can be obtained as follows:

(12) $$r_{c}\;=\;\left[\begin{array}{c} r_{c}^{1}\\ r_{c}^{2}\\ \vdots \\ r_{c}^{d} \end{array}\right]\;=\;\left[\begin{array}{ll} r_{a} & r_{b} \end{array}\right]\;=\;\left[\begin{array}{cc} r_{a}^{1} & r_{b}^{1}\\ r_{a}^{2} & r_{b}^{2}\\ \vdots & \vdots \\ r_{a}^{d} & r_{b}^{d} \end{array}\right]$$

where *d* is the dimensionality of the MMOPs. The terms *r_a_* and $r_{b}$ are fuzzily related by Equation (10), so the crossover operation of Equation (9) is referred to as the fuzzy crossover operation. The control parameters in *r_c_* can be used by a *d*-dimensional individual in a complete mutation and crossover operation.

### 3.3. Updated Parameters of the Bivariate Joint Normal Distribution

To improve the diversity of $r_{a}$ and *r_b_*, it is necessary to update *μ* and ∑ before each iteration of FSGWO.

#### 3.3.1. Update of *μ*

Updating *μ* with a fuzzy perturbation is described as follows.

$X_{p}$ before and after the update is denoted by $X_{p}^{old}$ and $X_{p}^{new}$, respectively. The fitness values of $X_{p}^{old}$ and $X_{p}^{new}$ are denoted by *f*($X_{p}^{old}$) and *f*($X_{p}^{new}$), respectively. The absolute value |*f*($X_{p}^{old}$) − *f*($X_{p}^{new}$)| represents the change rate of the fitness value before and after the update of $X_{p}$. In population *X*, the individual with the largest |*f*($X_{p}^{old}$) − *f*($X_{p}^{new}$)| is denoted by $X_{m}$, where *m* is the ID of *X_m_*. $X_{m}$ can be written as

(13) $$X_{m}\;=\;\underset{X_{p}\in X}{\arg\max}\left|f\left(X_{p}^{old}\right)\;-\;f\left(X_{p}^{new}\right)\right|$$

The control parameters of *X_m_* are stored in the *m*th row of $r_{c}$, denoted by *r^m^_c_* = [$r_{a}^{m}$,$r_{b}^{m}$]. $r_{c}^{m}$ can be regarded as heuristic information for updating *μ*, namely, fuzzy perturbation. Updating *μ* with $r_{c}^{m}$ can be written as

(14) $$\mu \;=\;(1\;-\;c)\;\times \;\mu \;+\;c\;\times \;r_{c}^{m}$$

where *c* is a conversion factor, which is a constant between (0, 1). The expression *c* × $r_{c}^{m}$ takes part of $r_{c}^{m}$ as heuristic information to update *μ*. To avoid excessive perturbation and cause the algorithm to diverge, *c* is usually 0.1 or 0.2.

#### 3.3.2. Update of ∑

The updated method of *∑* is relatively simple. First, $s_{1}$ is obtained by sampling the standard uniform distribution; *s*_2_ and $s_{3}$ are sampled via the standard normal distribution. Finally, a new *∑* can be obtained by substituting *s*_1_, $s_{2}$, and *s*_3_ into Equation (11).

### 3.4. Steps of FSGWO

The steps of FSGWO are shown in Algorithm 2.
**Algorithm 2.** Steps of the FSGWO algorithm.**Input:** grey wolf population size *M*, maximum number of iterations *T*, dimension *d* of the problem, upper bound *ub* and lower bound *lb* of variables.**Output:** optimal solution $X_{\alpha }$.1:Initialize the wolf population *X*, parameters *c*, *μ*, and *∑*.2:Calculate the fitness value of *f*(*X_p_*) for each grey wolf. The fitness of the entire population is denoted by *f*(*X^old^*).*f*(*X^new^*) = *f*(*X^old^*).3:Initialize the three leading wolves $X_{\alpha }$, *X_β_*, and $X_{\delta }$.4:for *t* = 1 to *T*4.1:Generate the control parameters matrix *r_c_* with Equation (12).4.2:for *p* = 1 to *M*4.2.1:Generate the new mutant individual $X_{\nu }$ with Equation (8).4.2.2:Generate *X_u_* with Equation (9); then randomly specify a dimension *ω*, and perform a crossover operation $X_{u}^{\omega }$ = $X_{\nu }^{\omega }$ to produce a new individual *X_u_*.4.2.3:Calculate the fitness value *f*(*X_u_*) of *X_u_*.4.2.4:If *f*(*X_u_*) < *f*($X_{p}$)   *X_p_* = *X_u_*.   In *f*(*X^new^*), *f*($X_{p}$) = *f*(*X_u_*).End if4.2.5:End for4.3:According to *f*(*X^new^*), update *X_α_*, $X_{\beta }$, and *X_δ_*.4.4:Calculate $X_{m}$ with Equation (13) and then obtain $r_{c}^{m}$ from $r_{c}$.Update *μ* and *∑* with Equation (14) and Equation (11).4.5:*f*(*X^old^*) = *f*(*X^new^*).4.6:End for5:Output optimal solution *X_α_*.

Some details of the algorithm steps in Algorithm 2 are described below.

(i) In step 1, the initial value of *μ* is [0.5, 0.5], and the initial value of *∑* is [0.1, 0; 0, 0.1]. The value of *c* is 0.1 or 0.2, which means taking 10% or 20% of $r_{c}^{m}$ as a fuzzy perturbation.
(ii) In step 4.1, the values of the elements in $r_{c}$, which are sampled from the binary joint normal distribution *N*(*μ*, *∑*), may be out of (0, 1); the element’s value is corrected to 0.999 if it crosses the upper bound or 0.001 if it crosses the lower bound.
(iii) In steps 4.2.1 and 4.2.2, the value of each element in *Xν* and *X_u_* is between [*lb*, *ub*]; if the value of an element exceeds the upper bound, it is corrected to *rand* × *ub*, or *rand* × *lb* if the value of an element exceeds the lower bound, where *rand* is a random number following a standard uniform distribution.
(iv) In step 4.4, the value of the element in *μ* is between (0, 1); if the value of an element in *μ* exceeds the upper bound, it is corrected to 0.99, or 0.01 if the value of an element exceeds the lower bound.
(v) Comparing the FSGWO algorithm flow in Algorithm 2 with the original GWO algorithm flow in Algorithm 1 shows that the operations lacking in Algorithm 1 mainly include fuzzy control parameters in step 4.1, the fuzzy crossover operation in step 3, noninferior selection in step 4.2.4, and fuzzy perturbation in step 4.4.

### 3.5. Analysis of Computational Complexity

*M* is the population size, *d* is the dimension of the problem, and *T* is the number of iterations.

According to the algorithm steps shown in Algorithm 2, the computational cost of FSGWO is concentrated in the iterative part. The computational cost of an iteration mainly includes the population mutant of *O*(*M* × *d*), the population crossover of *O*(*M* × *d*), the calculation of population fitness of *O*(*M*), an update of the leading wolfs of *O*(*M*), and an update of parameters of *O*(1). The computational cost of *T* iterations is *O*(*T* × (*M* × *d* + *M* × *d* + *M* + *M* + 1)). Therefore, the computational complexity of FSGWO is *O*(*T* × *M* × *d*).

According to the algorithm steps shown in Algorithm 1, the computational cost of the original GWO is concentrated in the iterative part. The computational cost of an iteration mainly includes the population mutant guided by the three leading wolves of *O*(*M* × *d* + *M* × *d* + *M* × *d*), the calculation of the population fitness of *O*(*M*), an update of the leading wolfs of *O*(*M*), and an update of the parameters of *O*(1). The computational cost of *T* iterations is *O*(*T* × (3 × *M* × *d* + 2 × *M* + 1)). Therefore, the computational complexity of the original GWO is *O*(*T* × *M* × *d*).

According to the above analysis, the computational complexity of FSGWO is the same as that of GWO.

## 4. Results

In this section, the FSGWO algorithm is verified on 30 test functions of IEEE CEC2014 [46] and 5 engineering application problems.

The compared algorithms include GWO [21], HPSOGWO [47], SOGWO [36], EO [48], and MPSO [49]. GWO is the original GWO. HPSOGWO is an improved GWO with a particle swarm individual update strategy. SOGWO uses selective opposition to enhance the diversity of GWO, and the convergence speed is faster. EO is inspired by control volume mass balance models used to estimate both dynamic and equilibrium states. MPSO is a particle swarm optimization algorithm using chaotic nonlinear inertia weights and has a good balance of diversity and convergence.

The key parameters of the competitive algorithms are shown in Table 1, and the computer source codes of those algorithms were provided by the original papers. The parameter values of the competition algorithm were taken from the original paper and the default settings of the source codes. The setting method of the control parameter values of FSGWO is described in Algorithm 2. In Table 1, *N* is the population size, which is uniformly taken as 50 in this paper. In EO, *a*_1_ is a constant value that controls exploration ability and *a*_2_ is a constant value used to manage exploitation ability; GP is a parameter used to balance exploration and exploitation. In MPSO, *c*_1_ and *c*_2_ are referred to as the acceleration factors; *ω*_1_ and *ω*_2_ are inertia weights used to balance exploration and exploitation. In GWO, parameter *a* is the neighborhood radius. In HPSOGWO, *rand* is a random number between (0, 1) and *ω* is an inertia weight. In SOGWO, parameter *a* is the neighborhood radius. In FSGWO, *c* is a conversion factor between (0, 1); $r_{a}$ and *r_b_* are adaptive control parameters.

### 4.1. Results of the Test Functions of CEC2014

The IEEE Congress on Evolutionary Computation 2014 (CEC2014) test suite had 30 complex optimization functions [46], where F1–F3 were unimodal functions and F4–F30 were multimodal functions. In this paper, the proposed algorithm was verified on 30 complex functions (F1–F30) of CEC2014. According to the requirements of the competition, the value range of each dimension decision variable was [−100, 100], and the maximum number of computations of the fitness function was d*10^4^. The experiment was repeated 51 times. The absolute value |*f*(*x*) − *f*(x*)| was the final result of a calculation, where *f*(*x*) was the optimal value of the function obtained by the algorithm, and *f*(x*) was the theoretical optimal value of the function. The smaller the value of |*f*(*x*) − *f*(x*)| was, the closer the optimal value obtained by the algorithm was to the theoretical optimal value. If |*f*(*x*) − *f*(x*)| < 10^−8^, the calculation result was 0.

#### 4.1.1. Results of 30-Dimensional Test Functions

Table 2 shows the calculation results of the related algorithms, in which the mean (Mean) and standard deviation (STD) of the index were calculated using the results of 51 runs of each algorithm. In Table 2, the optimal mean and standard deviation for each function are highlighted with a bold font and gray background.

From the mean results in Table 2, the calculated results of FSGWO on unimodal functions F1–F3 were at least four orders of magnitude better than those of GWO, HPSOGWO, and SOGWO. For the F1 function, the exponent of the result of FSGWO was e + 03, while the exponents of the results of GWO, HPSOGWO, and SOGWO were all e + 07. For the F2 function, the exponent of the result of FSGWO was e + 00, while the exponents of the results of GWO, HPSOGWO, and SOGWO were e + 09, e + 09, and e + 08. For the F3 function, the exponent of the result of FSGWO was e + 00, while the exponents of the results of GWO, HPSOGWO, and SOGWO were all e + 04. The results of the unimodal function show that FSGWO had excellent local optimization ability.

From the mean in Table 2, it can be seen that the calculation results of the proposed algorithm for 24 functions (F1–F5, F8–F12, F14, F16–F23, and F26–F30) were better than those of the competitive algorithms, accounting for 80.0%, which indicates that FSGWO could obtain high-quality solutions for MMOPs.

From the STD in Table 2, it can be seen that the results of the proposed algorithm for 23 functions (F1–F5, F8–F12, F14, F16–F23, F26, and F28–F30) were better than those of the competitive algorithms, accounting for 76.7%, which indicates that the FSGWO algorithm had good stability and convergence.

The mean results in Table 2 were analyzed by the Wilcoxon signed-rank test, and the significance level was 0.05. The results of the Wilcoxon test are shown in Table 3. In Table 3, all *p* values were less than 0.05, which meant that the mean values of FSGWO were significantly different from those of the other algorithms. The results of Table 2 and Table 3 show that the quality of solutions obtained by FSGWO was better than that of the competitive algorithms for the 30 30D test functions.

According to the data in Table 2, the percentage of improvement in calculation accuracy between FSGWO and each competing algorithm can be calculated, and the results are shown in Table 4. In Table 4, a negative number means that the calculation accuracy of FSGWO for this test function was not as good as that of the competitive algorithm and the term Average represents the average percentage of improvement in the calculation accuracy of FSGWO over the competitive algorithm for the 30 test functions. Table 4 shows that for the 30 30D test functions, the average calculation accuracy of FSGWO was 46.98%, 54.35%, 64.84%, 69.02%, and 62.27% higher than those of EO, MPSO, GWO, HPSOGWO, and SOGWO, respectively. The calculation accuracy of FSGWO was significantly higher than those of the competitive algorithms.

**Figure 2:**
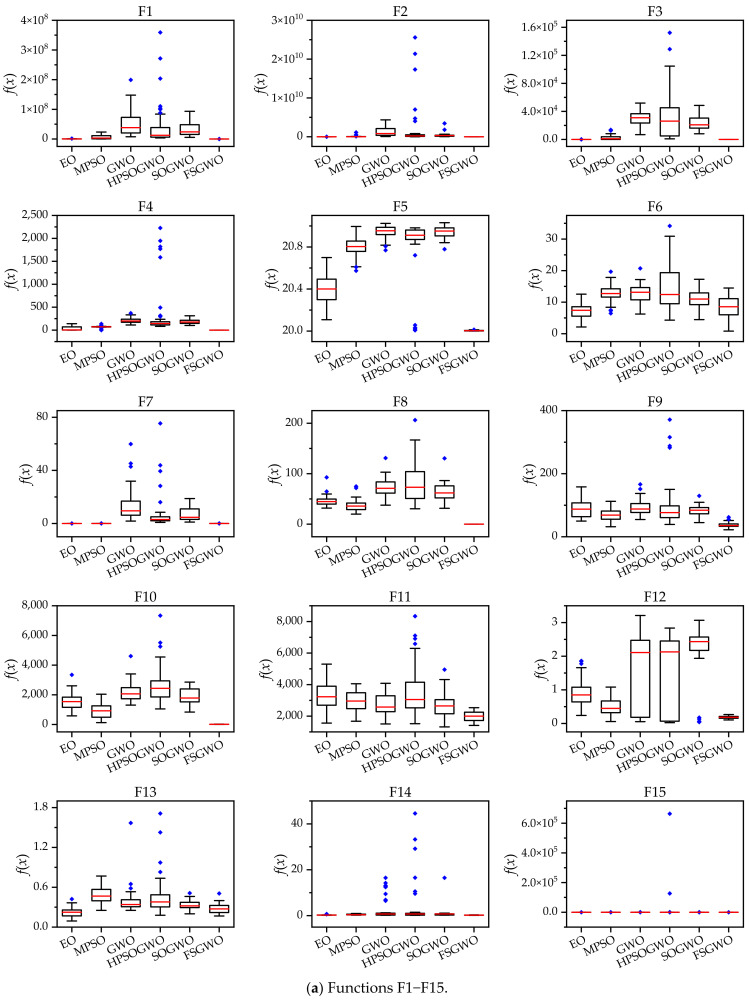

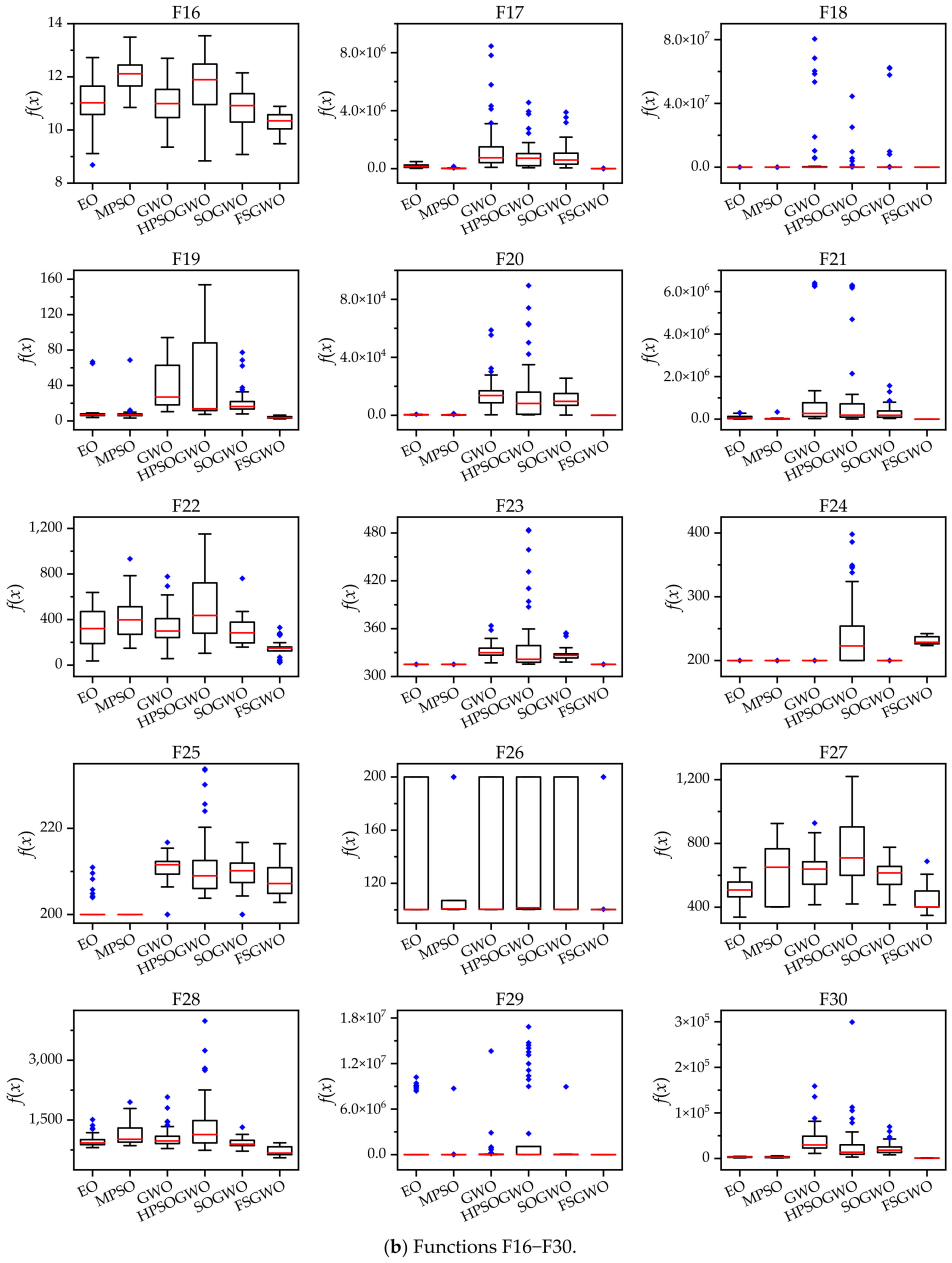
The box plots of the related algorithms for the 30-dimensional functions.

Figure 2 shows box plots of the related algorithms for the 30 test functions which were drawn with 51 calculation results of related algorithms. In Figure 2, the short red line represents the median; the black box represents the upper quartile (Q3) and the lower quartile (Q1); and the blue solid prism represents an outlier.

For 27 functions (F1–F5, F7–F23, and F26–F30), the box length of FSGWO was shorter than those of the competitive algorithms or comparable to them, which meant that the proposed algorithm had good convergence; therefore, the results of 51 runs were relatively concentrated, and the box length was shorter.

For 28 functions (F1–F23 and F26–F30), the median of the proposed algorithm was smaller than those of the competitive algorithms or comparable to them, which indicated that the proposed algorithm had good diversity and local optimization ability and could find high-quality solutions.

For 28 functions (F1–F25 and F28–F30), the number of outliers of FSGWO was less than those of the compared algorithms, or the outliers were mainly distributed around the median, which indicated that the calculation results of FSGWO were close to the normal distribution, and the robustness of FSGWO was better than those of the compared algorithms.

**Figure 3:**
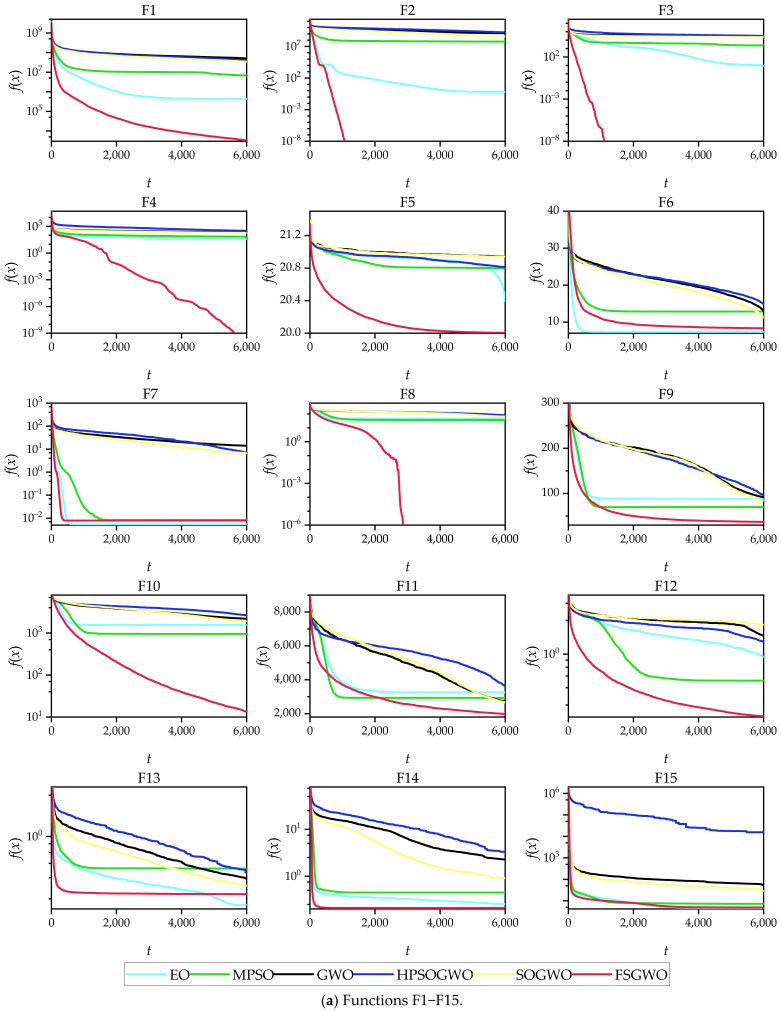

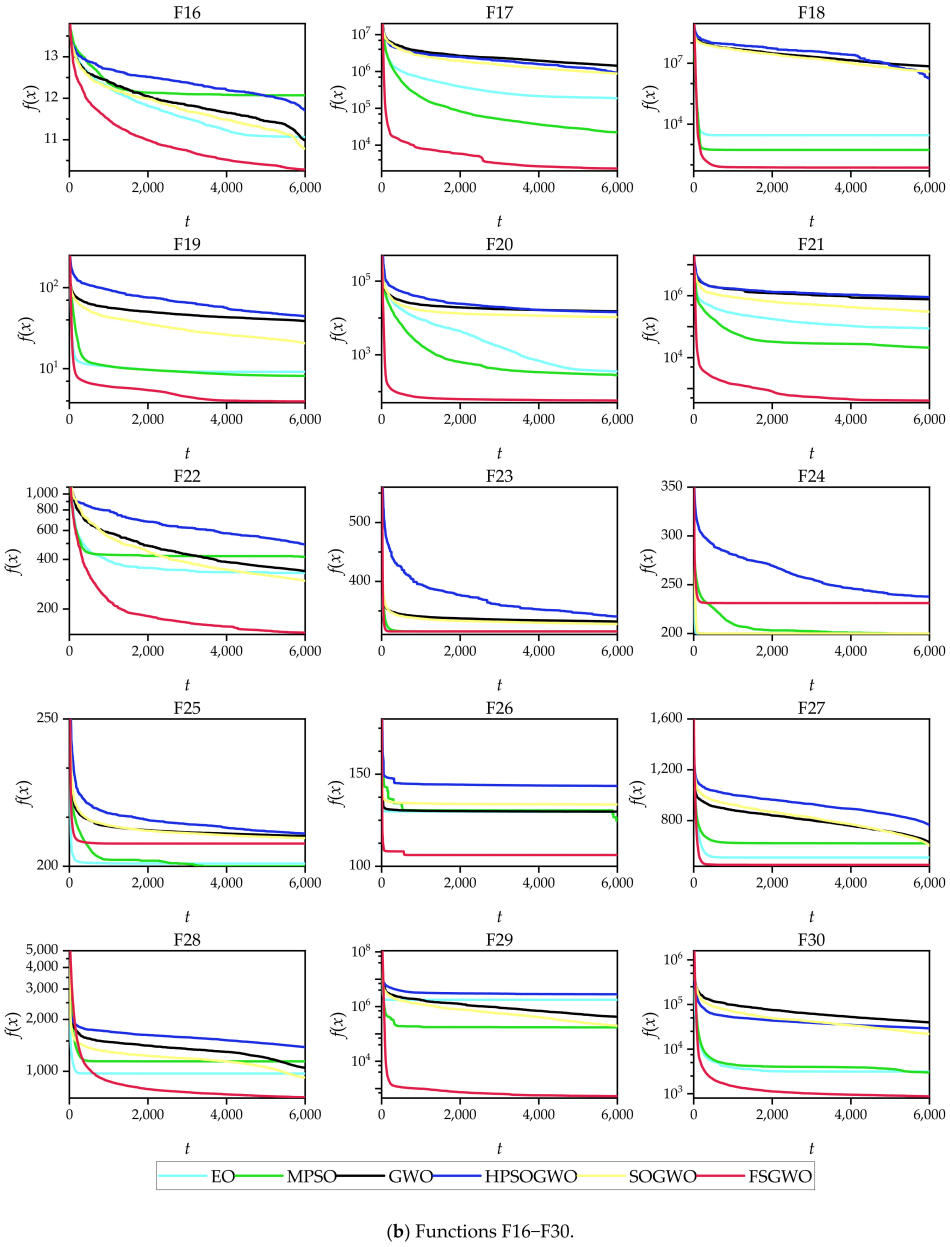
Convergence curves of the related algorithms for the 30-dimensional functions.

Figure 3 shows the convergence curves of the related algorithms for the 30 30D test functions, where the abscissa *t* is the number of iterations, and the ordinate *f*(*x*) is the average fitness of 51 independent experiments of each algorithm.

From the perspective of the changing tendencies of the convergence curves in the early stage of the iterations, the fitness values of the proposed algorithm decreased faster than those of the competitive algorithms for 29 functions (F1–F5 and F7–F30), which indicated that FSGWO had good diversity and convergence and could quickly locate the optimal solution in the early stage of the iterations.

From the overall change tendencies of the curves and the final convergence positions, for the 25 functions (F1–F5, F8–F12, F14–F23, and F26–F30), the convergence curves of FSGWO were better than those of the competitive algorithms.

#### 4.1.2. Results of 50-Dimensional Test Functions

The IEEE CEC2014 test functions were set to 50 dimensions. Table 5 shows the calculation results of the related algorithms for the 50-dimensional functions. In Table 5, the optimal mean and standard deviation for each function are highlighted with a bold font and gray background. As the dimensions increased, so did the complexity of the MMOPs. For 23 functions (F1–F5, F7–F12, F14, F16–F18, F20–F23, F26, and F28–F30), the mean values of FSGWO were better than those of the competitive algorithms, accounting for 76.7%.

The Wilcoxon signed-rank test was used to analyze the mean values in Table 5, with a significance level of 0.05. The results of the test are shown in Table 6. Table 6 shows that all *p* values were less than 0.05, which meant that the calculation results of FSGWO for the 50-dimensional functions were significantly different from those of the competitive algorithms.

According to the data in Table 5, the percentage of improvement in calculation accuracy between FSGWO and each competing algorithm can be calculated, and the results are shown in Table 7. In Table 7, a negative number means that the calculation accuracy of FSGWO for this test function was not as good as that of the competitive algorithm and the term Average represents the average percentage of improvement in the calculation accuracy of FSGWO over the competitive algorithm for 30 test functions. Table 7 shows that for the 30 50-dimensional test functions, the average calculation accuracy of FSGWO was 33.63%, 46.45%, 62.94%, 64.99%, and 59.82% higher than those of EO, MPSO, GWO, HPSOGWO, and SOGWO, respectively. The calculation accuracy of FSGWO was significantly higher than those of the competitive algorithms for the 50D test functions.

**Figure 4:**
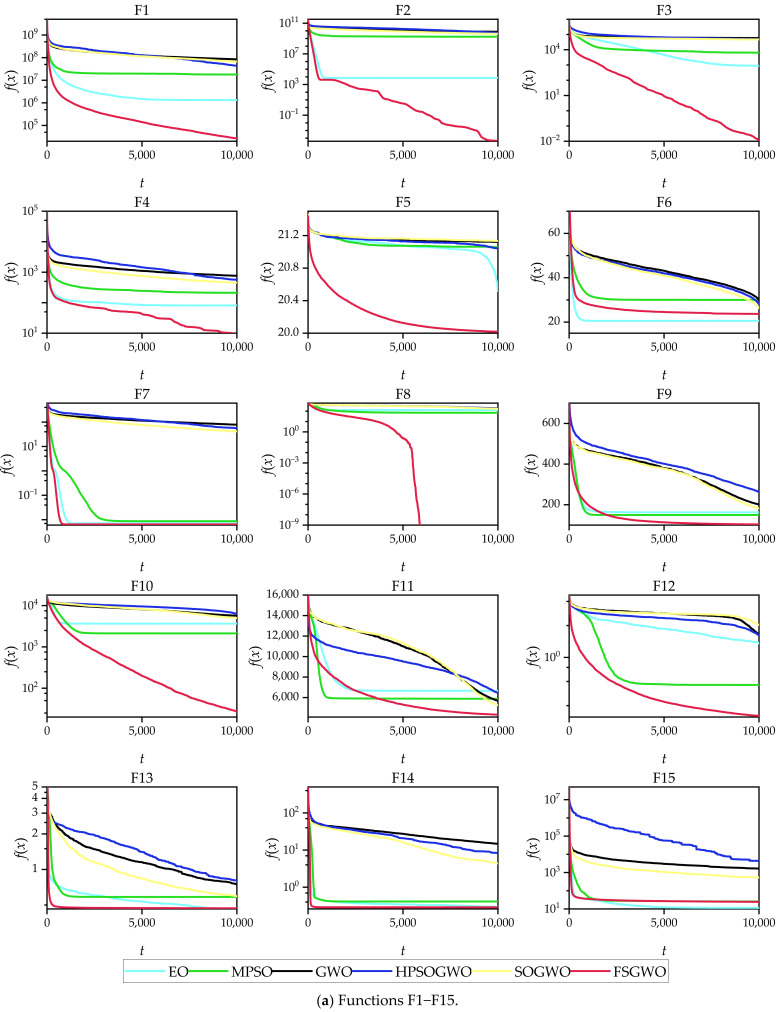

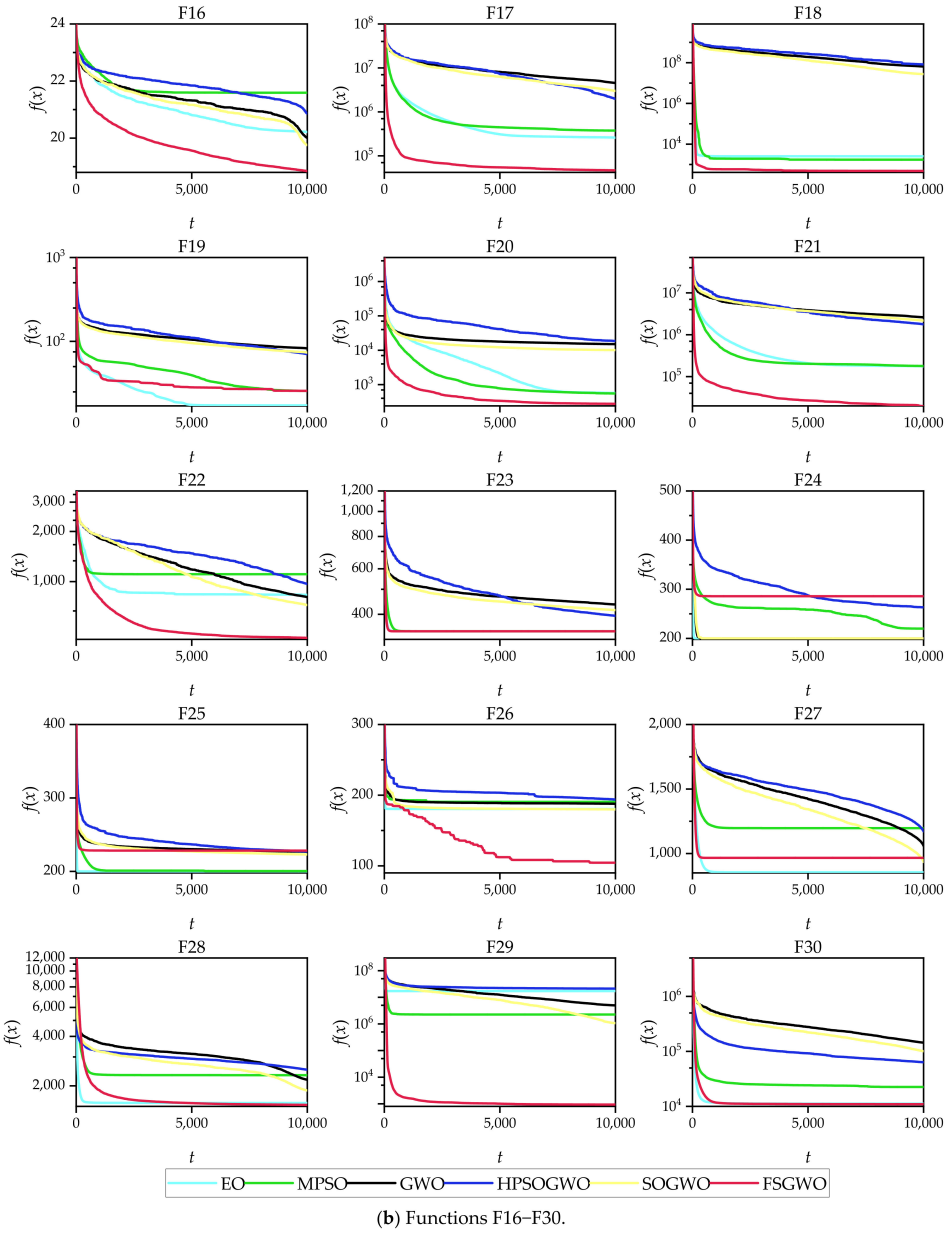
Convergence curves of the related algorithms for 50-dimensional functions.

Figure 4 shows the convergence curves of the related algorithms for the 50-dimensional functions, which were plotted with an average of 51 calculations of each algorithm. From the changing tendencies of the convergence curves and the final convergence positions, the convergence tendencies of the proposed algorithm for 25 test functions (F1–F5, F7–F14, F16–F18, F20–F23, and F26–F30) were better than those of the competitive algorithms.

#### 4.1.3. Verification of the Validity of the Fuzzy Control Parameters

In this paper, the correlation between control parameters $r_{a}$ and *r_b_* is modeled by the bivariate joint normal distribution *N*(*μ*, *∑*). A comparative experiment was conducted to verify the effectiveness of that modeling idea. The control parameters $r_{a}$ and *r_b_* were assumed to be independent random variables. In addition, both parameters followed the standard uniform distribution; the other parts of the FSGWO algorithm remained unchanged, and the new FSGWO at this time was denoted as FSGWO1.

The 30-dimensional functions (F1–F30) were solved by FSGWO and FSGWO1, and the calculation results are shown in Table 8. In Table 8, the optimal mean and standard deviation for each function are highlighted with a bold font and gray background. For 24 complex multimodal functions (F1–F5, F7–F12, F14–F16, F18, F19, F21–F23, F25, F26, and F28–F30), the mean values of FSGWO were better than those of FSGWO1, accounting for 80.0%. The Wilcoxon signed-rank test was used to analyze the mean values in Table 8, and the significance level was 0.05. The results of the test are shown in Table 9. The *p* value (2.7610e – 03) was less than the significance level, indicating that there was a substantial difference in Mean between FSGWO and FSGWO1.

**Figure 5:**
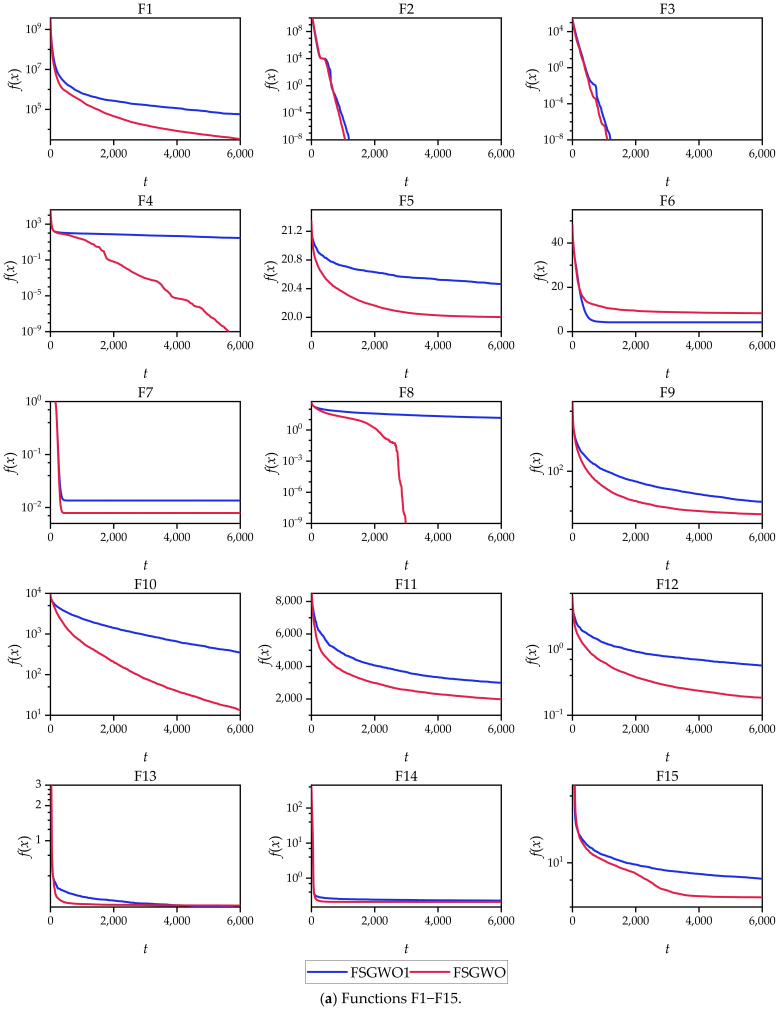

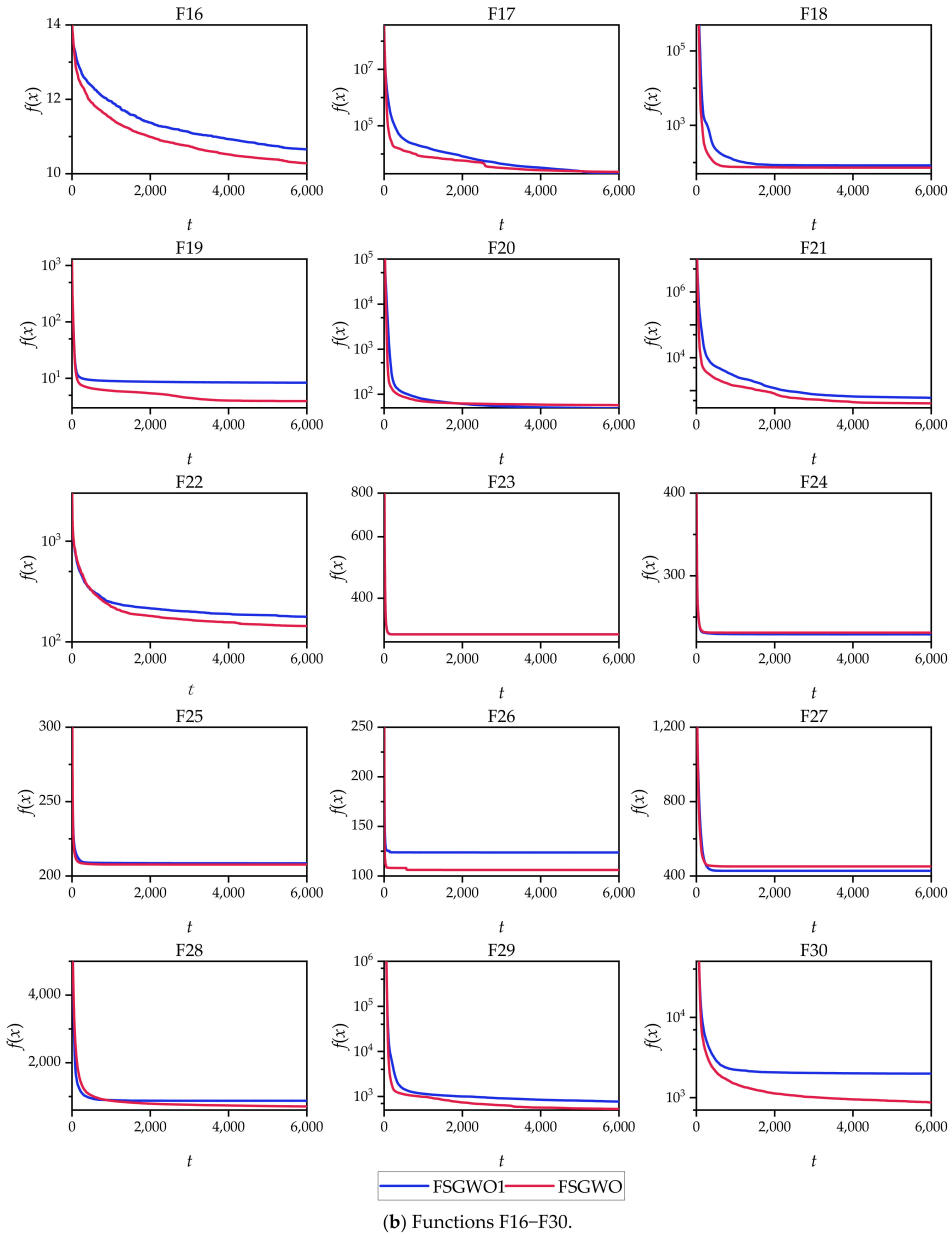
Convergence curves of FSGWO and FSGWO1.

Figure 5 demonstrates the convergence curves of FSGWO and FSGWO1 for the 30 30D test functions, which were plotted with the averages of 51 runs of the 2 algorithms. Figure 5 shows that the change tendencies and the final convergence positions of FSGWO were better than or comparable to those of FSGWO1 for 29 functions (F1–F5 and F7–F30).

According to the results of Table 8, Table 9 and Figure 5, the optimization ability of FSGWO was better than that of FSGWO1. Therefore, it is valid that binary joint normal distribution is used as a fuzzy method to realize the adaptive adjustment of the control parameters $r_{a}$ and *r_b_*, and this fuzzy method helps improve the convergence speed of FSGWO and the quality of the solution.

#### 4.1.4. Verification of the Effectiveness of the Fuzzy Perturbation Strategy

In Equation (14), the fuzzy perturbation $r_{c}^{m}$ is used to update the mean *μ* of the bivariate joint normal distribution. A comparative experiment was used to verify that $r_{c}^{m}$ was an effective design. $r_{c}^{m}$ was extracted from $r_{c}$ by *m* of Equation (13), and *m* was randomly generated instead of using Equation (13); the other parts of FSGWO remained unchanged, and the new FSGWO was denoted as FSGWO2.

The 30-dimensional functions were solved with FSGWO and FSGWO2, and the results are shown in Table 10. In Table 10, the optimal mean and standard deviation for each function are highlighted with a bold font and gray background. The mean values of FSGWO were better than those of FSGWO2 for 24 functions (F1–F5, F7–F12, F14, F15, F17–F19, F21–F24, F26, and F28–F30), accounting for 80.0%. Table 11 is the result of the Wilcoxon test for the mean values of Table 10, and the significance level was 0.05. The *p* value was 2.9719e-03, which was less than the significance level. The results of the Wilcoxon test indicated that there was a significant difference in the mean between FSGWO and FSGWO2.

**Figure 6:**
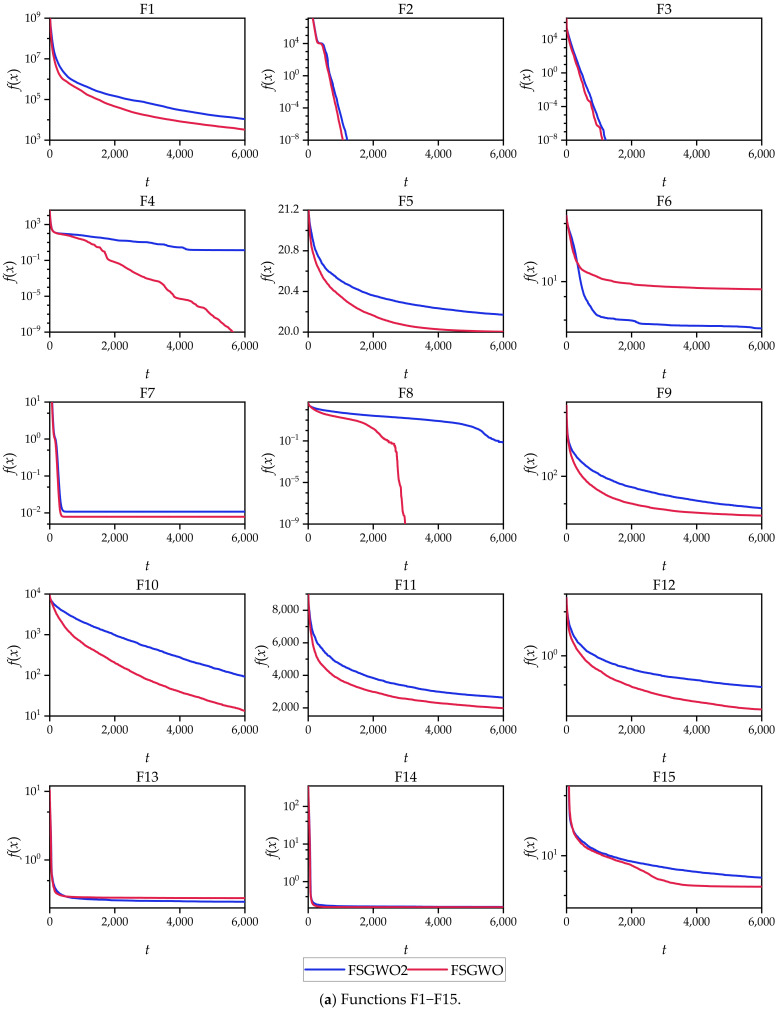

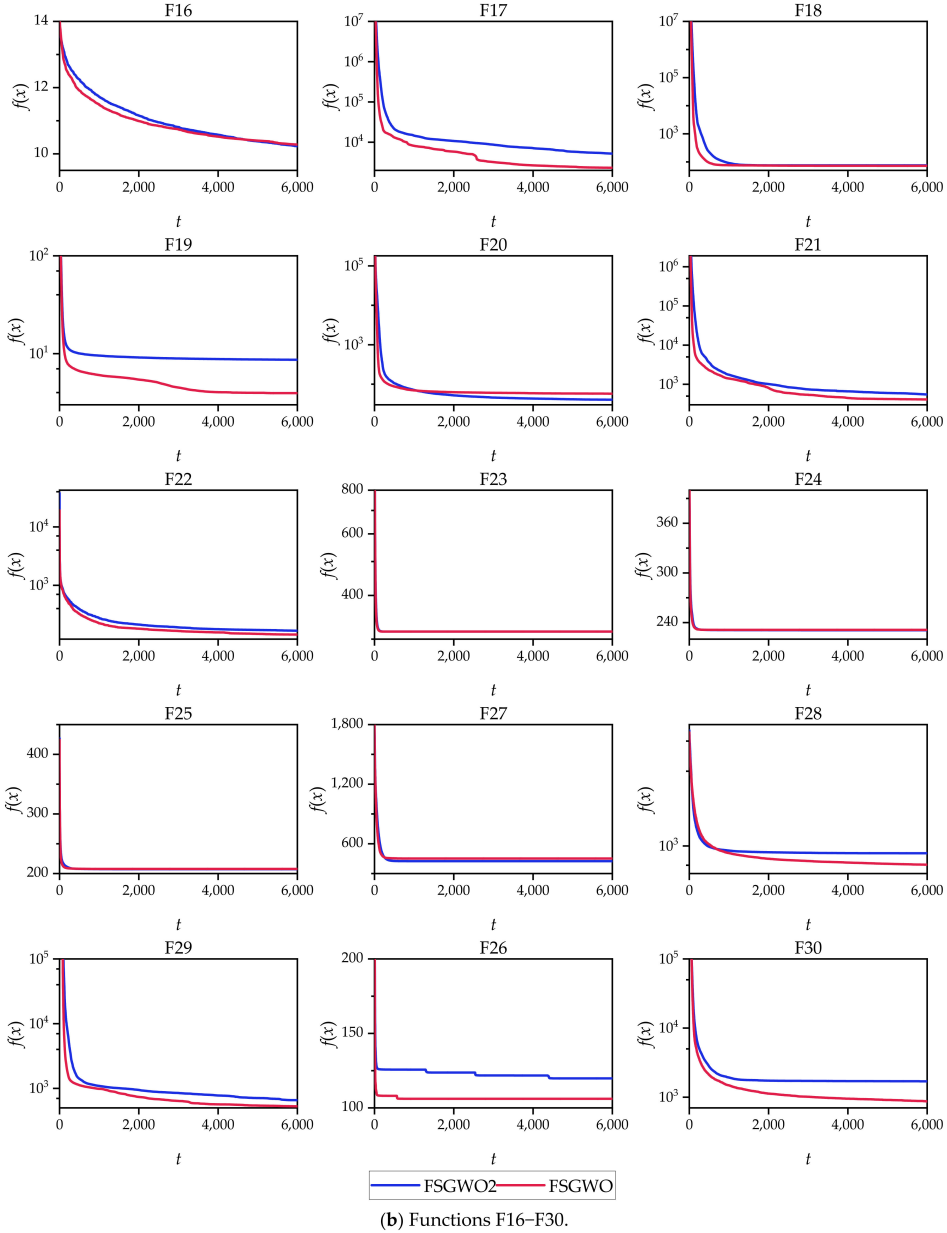
Convergence curves for FSGWO and FSGWO2.

Figure 6 shows the convergence curves of FSGWO and FSGWO2, which were plotted with the averages of 51 runs of the 2 algorithms. Figure 6 shows that FSGWO converged faster than FSGWO2 or was comparable to FSGWO2 for 29 functions (F1–F5 and F7–F30), accounting for 96.7%.

The results in Table 10 and Table 11 and Figure 6 show that the design idea of fuzzy perturbation is effective.

### 4.2. Results for Economic Load Dispatch Problems of Power Systems

Economic load dispatch (ELD) is a complex optimization problem with constraints in power systems [50,51]. The task of ELD is to reasonably dispatch the load of the system to each generator to minimize the fuel cost of the system and satisfy the relevant constraints.

#### 4.2.1. The Basic Model of the ELD Problem

The economic load dispatch of thermal power units is discussed in this paper. The fuel cost of an ELD can be approximately expressed as

(15) $$\min\;F\;=\;\sum_{i=1}^{N_{G}}F_{i}\left(P_{i}\right)\;=\;\sum_{i=1}^{N_{G}}\left(a_{i}P_{i}^{2}\;+\;b_{i}P_{i}\;+\;c_{i}\;+\;\left|e_{i}\;\times \;\sin\left(f_{i}\;\times \;\left(P_{i}^{\min}-P_{i}\right)\right)\right|\right)$$

where *i* is the ID of a generator unit and *N_G_* is the total number of generators, which is the dimension of the ELD. For the *i*th unit, $P_{i}^{\min}$ is the output power, $P_{i}$ is the minimum output power, $F_{i}$*(P_i_)* is the generation cost function, and $a_{i}$, *b_i_*, $c_{i}$, *e_i_* and $f_{i}$ are the power generation cost coefficients. The absolute value operator |·| converts the negative domain of the sine function into a positive domain to generate multimodality; thus, the ELD is a constrained high-dimensional multimodal optimization problem.

The main constraints of ELDs are as follows.

(i) Power balance constraints.

(16) $$\sum_{i\;=\;1}^{N_{G}}P_{i}\;=\;P_{D}\;+\;P_{L}$$

These constraints require that the total power generation of each unit is equal to the system load *P_D_* and the transmission loss $P_{L}$.

(ii) Generating capacity constraints.

(17) $$P_{i}^{\min}\;\leq \;P_{i}\;\leq \;P_{i}^{\max}$$

where *P^min^_i_* and $P_{i}^{max}$ are the lower and upper limits of the output power of the *i*th unit, respectively. These constraints require that $P_{i}$ is between [$P_{i}^{min}$, $P_{i}^{max}$].

(iii) Ramp rate limits.

(18) $$-\Delta P_{i}\;\leq \;P_{i}^{t}\;-\;P_{i}^{t-1}\;\leq \;\Delta P_{i}$$

where $P_{i}^{t-1}$ and $P_{i}^{t}$ are the output power of the *i*th unit in the (*t* − 1)th period and the *t*th period, respectively; Δ$P_{i}$ is the maximum change rate of the output power of the *i*th unit in the two adjacent periods. When multiperiod load dispatch is involved, |$P_{i}^{t-1}$ − $P_{i}^{t}$| cannot exceed Δ$P_{i}$.

- (iv) Prohibited operating zones.

(19) $$P_{i}\;\leq \;{\underline{P}}_{i}^{pz}\;and\;P_{i}\;\geq \;{\bar{P}}_{i}^{pz}$$

where ${\underline{P}}_{i}^{pz}$ and ${\bar{P}}_{i}^{pz}$ are the lower and upper limits of the prohibited operation zones of the *i*th unit, respectively. As there are physical limitations of generator components and unstable factors such as steam valve or bearing vibration, the output power of the *i*th unit is prohibited in some zones.

#### 4.2.2. Results of ELD Cases

The data of static ELD cases were taken from the IEEE CEC2011 competition dataset [51]; the numbers of units were 40 and 140, respectively. According to the requirements of the competition, the maximum number of calculations of the fitness function was 15,000. The best result (best), mean (mean), median (median), the worst result (worst), and standard deviation (STD) were calculated with 25 independent running results of the algorithm.

In addition to the six algorithms shown in Table 1, the compared algorithms included the island-based harmony search (iHS) [52], intellects-masses optimizer (IMO) [53], modified intellects-masses optimizer (MIMO) [53], adaptive population-based simplex (APS 9) [54], enhanced salp swarm algorithm (ESSA) [55], and the genetic algorithm with a new multiparent crossover (GA-MPC) [56]. iHS is a multipopulation evolutionary algorithm with an island-based harmony search. IMO is a dual-population culture algorithm, and its parameters hardly need to be adjusted. MIMP is an IMO algorithm with a trust domain reflection strategy and strong local search abilities. APS 9 is an improved adaptive population-based simplex method. ESSA is a multistrategy enhanced salp swarm algorithm. GA-MPC is a genetic algorithm with three consecutive parents.

Table 12 shows the results of the related algorithms for the 40-unit case. The results of the 1st–6th algorithms are calculated and presented in this paper, and the results of the 7th–12th algorithms were taken from the original papers. In Table 12, the best values of the indicators are highlighted with bold font and a gray background; the symbol—indicates that the data are not provided in the original paper.

From the mean index in Table 12, the exponents of the results of related algorithms were all e + 05, and the coefficients of the results were also very close, which meant that all algorithms could approximate the optimal solution; the nuance of the results was mainly caused by the different local optimization abilities of each algorithm. The Best, Mean, and Median of the FSGWO were 1.2260e + 05, 1.2514e + 05, and 1.2519e + 05, respectively, which were better than those of competitive algorithms, indicating that FSGWO had relatively strong local optimization ability and stability.

Table 13 shows the results of the related algorithms for the 140-unit case. In Table 13, the best values of the indicators are highlighted with a bold font and gray background. The best, mean, and median of FSGWO were 1.7551e + 06, 1.8119e + 06, and 1.8107e + 06, respectively, which were still better than those of the competitive algorithms, indicating that the proposed algorithm still had excellent optimization performance for the high-dimensional ELD problem. The simplex of ESSA and trust region of MIMO were both classical numerical optimization strategies; Table 13 shows that the fuzzy search strategy of FSGWO was competitive with those numerical optimization strategies when used to solve high-dimensional ELD problems.

According to the results of Table 12 and Table 13, when FSGWO was used to solve high-dimensional ELD problems, its optimization ability was better than those of the competitive algorithms, and higher-quality solutions could be obtained by FSGWO.

### 4.3. Design of Three-Bar Truss

**Figure 7:**
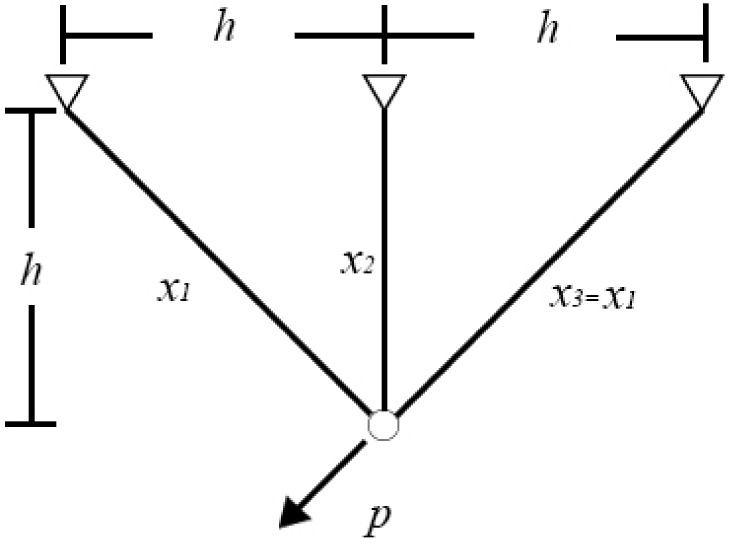
Three-bar truss design.

In structural engineering, a truss is a triangulated system that provides an efficient way to span long distances. Because members of a truss incur only axial force, the purpose of a truss design is to use less material and maintain the effectiveness of the entire system. A reduction in the amount of material used is usually expressed as a reduction in the diameter of a member. A three-bar planar truss structure is shown in Figure 7 [57]. In this problem, *x*_1_, *x*_2_, and *x*_3_ are the normalized diameters of the three members, and *x*_3_ has the same diameter as *x*_1_. The aim of this study is to achieve the minimum volume of a three-bar truss by minimizing the values of *x*_1_ and *x*_2_.

The problem shown in Figure 7 can be expressed as an optimization problem:

(20) $$\begin{array}{l} \min\;f(x_{1},x_{2})\;=\;(2\sqrt{2}x_{1}\;+\;x_{2})\;\times \;l\\ s.t.\left\{\begin{array}{l} \frac{\sqrt{2}x_{1}\;+\;x_{2}}{\sqrt{2}x_{1}^{2}\;+\;2x_{1}x_{2}}\;\times \;r\;-\;\rho \;\leq \;0\\ \frac{x_{2}}{\sqrt{2}x_{1}^{2}\;+\;2x_{1}x_{2}}\;\times \;r\;-\;\rho \;\leq \;0\\ \frac{1}{x_{1}\;+\;\sqrt{2}x_{2}}\;\times \;r\;-\;\rho \;\leq \;0 \end{array}\right.\\ l\;=\;100\mathrm{cm},r\;=\;2\mathrm{KN}/{\mathrm{cm}}^{2},\rho \;=\;2\mathrm{KN}/{\mathrm{cm}}^{2} \end{array}$$

where *x*_1_ and *x*_2_ are between [0, 1].

FSGWO was applied to solve the optimization problem of Equation (20). The competitive algorithms included the memory-based grey wolf optimizer (m-GWO) [58], modified sine cosine algorithm (m-SCA) [59], moth-flame optimization (MFO) [60], and cuckoo search (CS) [57]. Table 14 shows the results of related algorithms for the three-bar truss design problem. In Table 14, the best object function value is highlighted with a bold font and gray background. The data for the competitive algorithms are taken from the original papers. From Table 14, the objective function value of FSGWO is 263.8958, which is better than those of the competitive algorithms.

### 4.4. Design of Pressure Vessel

**Figure 8:**
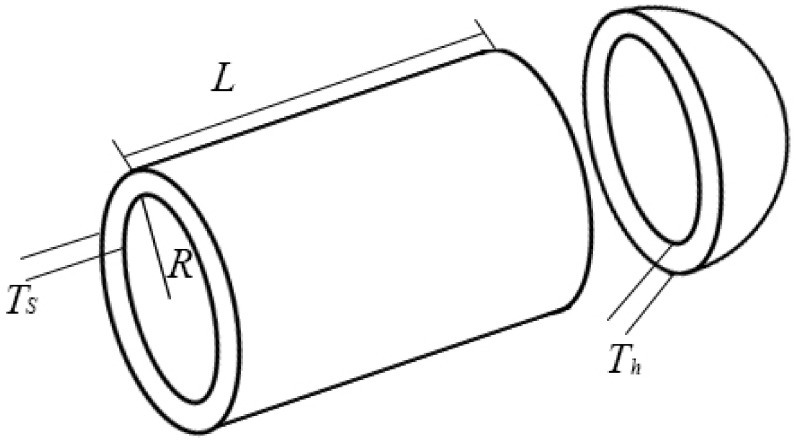
Pressure vessel design.

Figure 8 shows a cylindrical pressure vessel which has a hemispherical head at the end and is designed according to the ASME boiler and pressure vessel code [57]. This problem has four decision variables, which are the thickness of the shell (*Ts*), the thickness of the head (*Th*), the inner radius (*R*), and the length of the cylindrical section without considering the head (*L*). The goal of this problem is to minimize the cost of producing this capacity and satisfy the relevant conditions.

Four decision variables of the pressure vessel are represented by *x*_1_, *x*_2_, *x*_3_, and *x*_4_. The problem shown in Figure 8 can be expressed as an optimization problem:

(21) $$\begin{array}{c} \min\;f(x_{1},x_{2},x_{3},x_{4})\;=\;0.6224x_{1}x_{2}x_{4}\\ +\;1.7781x_{1}^{2}x_{3}\;+\;3.1661x_{1}^{2}x_{4}\;+\;19.84x_{1}^{2}x_{3}\\ s.t.\left\{\begin{array}{l} -\;x_{1}\;+\;0.0193x_{3}\;\leq \;0\\ -\;x_{2}\;+\;0.00954x_{3}\;\leq \;0\\ -\;\pi x_{3}^{2}x_{4}\;-\;\frac{4}{3}\pi x_{3}^{3}\;+\;1296000\;\leq \;0\\ x_{4}\;-\;240\;\leq \;0 \end{array}\right. \end{array}$$

where *x*_1_ and *x*_2_ are between [0, 99] and *x*_3_ and *x*_4_ are between [10, 200].

FSGWO was applied to solve the optimization problem of Equation (21). The competitive algorithms included the grey wolf optimization method based on a beetle antenna strategy (BGWO) [61], the improved grey wolf optimizer (I-GWO) [62], moth-flame optimization with orthogonal learning and Broyden-Fletcher-Goldfarb-Shanno (BFGSOLMFO) [63] and the slime mould algorithm (SMA) [64]. Table 15 shows the results of related algorithms for the pressure vessel problem. In Table 15, the best object function value is highlighted with a bold font and gray background. The data for the competitive algorithms are taken from the original papers. From Table 15, the objective function value of FSGWO is 5885.3328, which is better than those of the competitive algorithms.

### 4.5. Design of Gear Train

**Figure 9:**
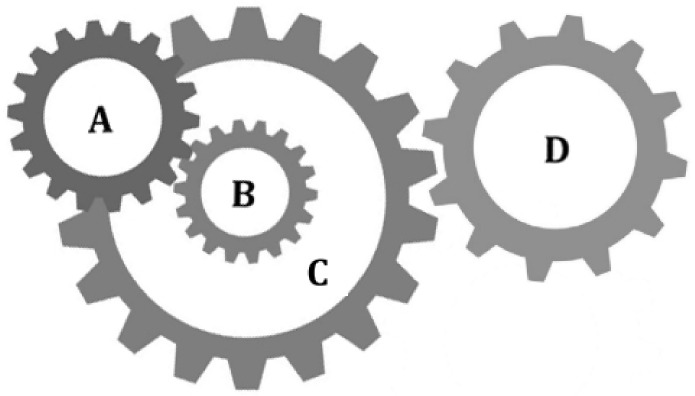
Gear train design.

Figure 9 shows a gear train design problem, in which there are four gears A, B, C and D [59]. The numbers of teeth of the four gears are represented by variables *x*_1_, *x*_2_, *x*_3_, and *x*_4_. The number of teeth is an integer between [12,60]. The goal of this problem is to minimize the gear ratio and keep it close to the optimal value of 1/6.931.

The problem of Figure 9 can be expressed as an optimization problem:

(22) $$\begin{array}{l} \min\;f(x_{1},x_{2},x_{3},x_{4})\;=\;{\left(\frac{1}{6.931}\;-\;\frac{x_{1}x_{3}}{x_{2}x_{4}}\right)}^{2}\\ s.t.\;12\;\leq \;x_{i}\;\leq \;60,\;\mathrm{and}\;x_{i}\;\in \;Z^{+}\;\forall i\;=\;1,2,3,4. \end{array}$$

FSGWO was applied to solve the optimization problem of Equation (22). The competitive algorithms included m-SCA [59], CS [57], the linear prediction evolution algorithm (LPE) [65], and the hybrid grey wolf optimizer and sine cosine algorithm (GWOSCA) [66]. Table 16 shows the results of related algorithms for the gear train design problem. In Table 16, the best object function values are highlighted with a bold font and gray background. The data for the competitive algorithms are taken from the original papers. From Table 16, the objective function value of FSGWO is 2.7009e−12, which is as good as those of m-SCA, CS, and LPE. Moreover, Table 16 shows that gear train design is a typical multimodal optimization problem. For the objective function value of 2.7009e−12, there are three different nearly optimal solutions that correspond to different gear train designs. According to the cost, volume, weight, and reliability of the gear train, decision makers find a design that meets their requirements among these different solutions.

### 4.6. Design of Cantilever Beam

**Figure 10:**
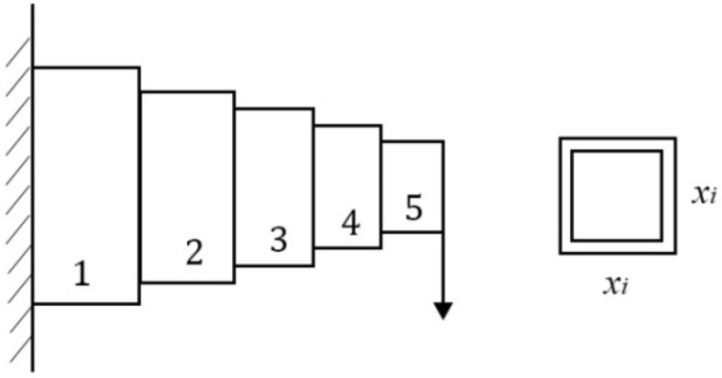
Cantilever beam design.

Figure 10 shows a cantilever beam design problem in which there are five nodes [59]. A node in Figure 10 is regarded as a square hollow cross-section with constant thickness. The first node is fixedly supported, and there is an external vertical force acting at the end of the fifth node. The variable *x_i_* represents the width of the cross-section of the *i*th node and its value is between [0.01, 100]. The goal of this problem is to minimize the weight of the cantilever beam.

The problem of Figure 10 can be expressed as an optimization problem:

(23) $$\begin{array}{l} \min\;f(x_{1},x_{2},x_{3},x_{4},x_{5})\;=\;0.0624\;\times \;\sum_{i=1}^{5}x_{i}\\ \begin{array}{cc} s.t. & \frac{61}{x_{1}^{3}} \end{array}\;+\;\frac{37}{x_{2}^{3}}\;+\;\frac{19}{x_{3}^{3}}\;+\;\frac{7}{x_{4}^{3}}\;+\;\frac{1}{x_{5}^{3}}\;\leq \;1\\ \;\;0.01\;\leq \;x_{i}\;\leq \;100,\;i\;=\;1,2,3,4,5 \end{array}$$

FSGWO was applied to solve the optimization problem of Equation (23). The competitive algorithms included CS [57], BGWO [61], m-SCA [59], and MFO [60]. Table 17 shows the results of related algorithms for the cantilever beam design problem. In Table 17, the best object function values are highlighted with a bold font and gray background. The data for the competitive algorithms are taken from the original papers. From Table 17, the objective function value of FSGWO is 1.33996, which is as good as that of BGWO.

## 5. Discussion

The convergence curves of Figure 3 and Figure 4 show that the fitness values of the FSGWO algorithm decreased faster than those of the competitive algorithms in the early stage of iterations. This advantage is related to the improvement of FSGWO in population updates. Step 4.2.4 in Algorithm 2 utilizes the noninferior selection strategy for population updating, which allows only better new individuals to be updated into the population. As the entire grey wolf population can be used to estimate the position of the optimal solution, the probability of detecting the region where the optimal solution is located is also increased, and FSGWO has a faster convergence speed in the early stage of iterations. In contrast, the traditional GWO uses only three leading wolves to estimate the region of the optimal solution, and the probability of finding the optimal solution is relatively low; thus, the convergence curve slowly decreases.

The results in Table 2, Table 3, Table 4, Table 5, Table 6, Table 7, Table 8, Table 9, Table 10, Table 11, Table 12, Table 13, Table 14, Table 15, Table 16 and Table 17 and Figure 2 show that the FSGWO algorithm can obtain higher-quality solutions, which indicates that the proposed algorithm has strong optimization ability and stability. These advantages of FSGWO come from the following improvements. (i) The fuzzy direction *D_c_* is added with both global and local search information, which enhances the ability of FSGWO to approximate the optimal solution, thereby producing a high-quality solution. (ii) The new individual *X_u_* generated by the fuzzy crossover operator does not mutate in all dimensions, which effectively controls the divergence of the algorithm and improves the stability and robustness of FSGWO. (iii) The binary joint normal distribution and fuzzy perturbation can adaptively adjust the control parameters $r_{a}$ and *r_b_* of FSGWO, which not only reduces the blindness of the selection of control parameters but also helps improve the local search ability and stability of FSGWO.

Other evolutionary algorithms also have control parameters, and the proposed modeling idea of control parameters is also suitable for those evolutionary algorithms. For example, an evolutionary algorithm has four control parameters, denoted as $r_{1}$, *r*_2_, $r_{3}$, and *r*_4_. The internal relation of these four control parameters can be modeled by a quaternary joint normal distribution *N*(*μ*, *∑*), and *μ* is written as

(24) $$\mu \;=\;\left[\mu_{1},\mu_{2},\mu_{3},\mu_{4}\right]$$

where $\mu_{1}$, *μ*_2_, $\mu_{3}$, and *μ*_4_ are scalars between (0, 1), and their initial values are 0.5. The covariance matrix *∑* can be expressed as

(25) $$\Sigma \;=\;\left[\begin{array}{cccc} s_{1}\;\times \;s_{2} & 0 & 0 & 0\\ 0 & s_{1}\;\times \;s_{3} & 0 & 0\\ 0 & 0 & s_{1}\;\times \;s_{4} & 0\\ 0 & 0 & 0 & s_{1}\;\times \;s_{5} \end{array}\right]$$

where $s_{1}$ is a random number following the standard uniform distribution. The four terms *s*_2_*–*$s_{5}$ are random numbers following the standard normal distribution. The initial values of $s_{1}$*–s*_5_ are all 0.1. A set of control parameters ($r_{1}$, *r*_2_, $r_{3}$, *r*_4_) can be obtained by sampling the quaternary joint normal distribution *N*(*μ*, *∑*). In the iterative process, the updated methods of *μ* and *∑* are the same as those in this study.

In summary, the experimental results show that the improvements of FSGWO in balancing diversity and convergence are feasible and effective. The proposed algorithm can produce high-quality solutions when used to solve high-dimensional complex MMOPs and has good convergence and stability.

## 6. Conclusions

To address the issue that the traditional GWO solves high-dimensional MMOPs with slow convergence speed and low quality solutions, a fuzzy strategy grey wolf optimizer (FSGWO) is proposed in this paper, the key improvements of which are as follows. (i) A new individual mutation strategy is proposed, which utilizes both global and local search information in the fuzzy search direction of mutation and enhances the ability of grey wolf individuals to find the optimal solutions. (ii) A fuzzy crossover operator is used to prevent new individuals from mutating in all dimensions and effectively improves the local search ability of FSGWO and the quality of solutions. (iii) The noninferior selection strategy is applied to update the population, and only better new individuals are allowed to update in the population. Therefore, the entire grey wolf population can be used to estimate the region where the optimal solution is located, which speeds up the convergence of FSGWO. (iv) The two control parameters of FSGWO are modeled by a binary joint normal distribution whose parameters are adaptively updated by a fuzzy perturbation, which effectively reduces the blindness of control parameter selection and improves the stability of the proposed algorithm. Finally, FSGWO is verified on 30 complex test functions of IEEE CEC2014 and 5 engineering application problems; the results show that the convergence of the proposed algorithm and quality of solutions are better than those of the competitive algorithms, which means that the improvements of FSGWO are feasible and effective.

Recent studies have shown that multiple populations have advantages over a single population in maintaining diversity and convergence [67,68]. For our future works, we are interested in some novel topics on GWOs with multiple populations, such as the exchange method of optimal solution information between different populations and the design idea of individual search direction in multiple populations. In addition, state-of-the-art evolutionary algorithms, such as self-adaptive quasi-oppositional stochastic fractal search [69] and combined social engineering particle swarm optimization [70], have many creative update strategies for populations and can be used for reference in the future improvement of FSGWO.

## Figures and Tables

**Table 1:** Key parameters of the competitive algorithms.

Algorithm	Parameters
EO	*N* = 50, *a_1_* = 2, *a_2_* = 1, *GP* = 0.5
MPSO	*N* = 50, *w_1_* = 0.9, *w_2_* = 0.4, *c_1_* = 2, *c_2_* = 2
GWO	*N* = 50, *a* = 2
HPSOGWO	*N* = 50, *w* = 0.5 + *rand*
SOGWO	*N* = 50, *a* = 2
FSGWO	*N* = 50, *c* = 0.2*, $r_{c}^{j}$*= [$r_{a}^{j}$, $r_{b}^{j}$] ~ *N*(*μ, ∑*)

**Table 2:** Results of the related algorithms for 30-dimensional test functions.

Function	Index	EO	MPSO	GWO	HPSOGWO	SOGWO	FSGWO
F1	Mean	4.30e + 05	6.80e + 06	5.05e + 07	4.01e + 07	3.43e + 07	**3.29e + 03**
	STD	2.80e + 05	7.50e + 06	4.11e + 07	6.88e + 07	2.36e + 07	**3.46e + 03**
F2	Mean	6.31e − 01	5.91e + 07	1.26e + 09	1.77e + 09	3.79e + 08	**0.00e + 00**
	STD	7.96e − 01	1.72e + 08	1.07e + 09	5.18e + 09	5.15e + 08	**0.00e + 00**
F3	Mean	8.97e + 00	2.36e + 03	3.11e + 04	3.26e + 04	2.28e + 04	**0.00e + 00**
	STD	9.08e + 00	3.23e + 03	9.49e + 03	3.33e + 04	9.80e + 03	**0.00e + 00**
F4	Mean	3.75e + 01	6.79e + 01	2.11e + 02	3.21e + 02	1.84e + 02	**0.00e + 00**
	STD	4.27e + 01	3.08e + 01	5.54e + 01	5.24e + 02	4.45e + 01	**0.00e + 00**
F5	Mean	2.04e + 01	2.08e + 01	2.09e + 01	2.08e + 01	2.09e + 01	**2.00e + 01**
	STD	1.25e − 01	9.25e − 02	5.86e − 02	2.94e − 01	5.46e − 02	**3.28e − 03**
F6	Mean	**7.35e + 00**	1.29e + 01	1.29e + 01	1.48e + 01	1.11e + 01	8.33e + 00
	STD	**2.58e + 00**	2.76e + 00	2.79e + 00	7.42e + 00	3.16e + 00	3.57e + 00
F7	Mean	**5.36e − 03**	8.34e − 03	1.41e + 01	6.93e + 00	6.70e + 00	7.86e − 03
	STD	**8.73e − 03**	1.43e − 02	1.20e + 01	1.30e + 01	4.77e + 00	1.21e − 02
F8	Mean	4.60e + 01	3.64e + 01	7.28e + 01	8.03e + 01	6.45e + 01	0.00e + 00
	STD	1.04e + 01	1.15e + 01	1.62e + 01	3.57e + 01	1.69e + 01	**0.00e + 00**
F9	Mean	8.84e + 01	6.99e + 01	9.19e + 01	9.65e + 01	8.34e + 01	**3.71e + 01**
	STD	2.74e + 01	1.83e + 01	2.37e + 01	6.92e + 01	1.57e + 01	**8.27e + 00**
F10	Mean	1.56e + 03	9.51e + 02	2.19e + 03	2.64e + 03	1.90e + 03	**1.35e + 01**
	STD	5.46e + 02	4.86e + 02	6.26e + 02	1.25e + 03	5.31e + 02	**3.73e + 00**
F11	Mean	3.26e + 03	2.93e + 03	2.73e + 03	3.64e + 03	2.67e + 03	**1.98e + 03**
	STD	7.43e + 02	6.43e + 02	6.29e + 02	1.67e + 03	7.22e + 02	**3.09e + 02**
F12	Mean	9.18e − 01	4.82e − 01	1.64e + 00	1.40e + 00	2.23e + 00	**1.84e − 01**
	STD	3.90e − 01	2.51e − 01	1.08e + 00	1.17e + 00	7.53e − 01	**3.67e − 02**
F13	Mean	**2.19e − 01**	4.90e − 01	3.89e − 01	4.47e − 01	3.33e − 01	2.78e − 01
	STD	6.95e − 02	1.10e − 01	1.87e − 01	2.79e − 01	**6.63e − 02**	6.68e − 02
F14	Mean	2.55e − 01	4.47e − 01	2.26e + 00	3.31e + 00	9.11e − 01	**2.10e − 01**
	STD	1.10e − 01	1.96e − 01	4.25e + 00	8.81e + 00	2.24e + 00	**4.68e − 02**
F15	Mean	**4.48e + 00**	7.28e + 00	5.38e + 01	1.55e + 04	2.88e + 01	4.89e + 00
	STD	**1.27e + 00**	3.12e + 00	1.07e + 02	9.43e + 04	5.52e + 01	1.31e + 00
F16	Mean	1.11e + 01	1.21e + 01	1.10e + 01	1.17e + 01	1.08e + 01	**1.03e + 01**
	STD	8.75e − 01	5.60e − 01	7.16e − 01	1.07e + 00	7.09e − 01	**3.73e − 01**
F17	Mean	1.87e + 05	2.24e + 04	1.43e + 06	9.24e + 05	8.78e + 05	**2.32e + 03**
	STD	1.27e + 05	2.40e + 04	1.81e + 06	9.99e + 05	8.59e + 05	**4.37e + 03**
F18	Mean	2.85e + 03	5.40e + 02	7.14e + 06	1.79e + 06	3.95e + 06	**7.26e + 01**
	STD	4.01e + 03	5.91e + 02	1.95e + 07	7.19e + 06	1.45e + 07	**3.08e + 01**
F19	Mean	9.13e + 00	8.14e + 00	3.87e + 01	4.43e + 01	2.06e + 01	**3.95e + 00**
	STD	1.16e + 01	8.81e + 00	2.53e + 01	5.07e + 01	1.39e + 01	**1.13e + 00**
F20	Mean	3.55e + 02	2.77e + 02	1.52e + 04	1.45e + 04	1.05e + 04	**5.73e + 01**
	STD	1.19e + 02	1.97e + 02	1.06e + 04	2.03e + 04	5.75e + 03	**3.14e + 01**
F21	Mean	8.76e + 04	2.10e + 04	7.68e + 05	8.95e + 05	3.03e + 05	**4.10e + 02**
	STD	8.12e + 04	4.71e + 04	1.46e + 06	1.74e + 06	3.25e + 05	**2.49e + 02**
F22	Mean	3.31e + 02	4.15e + 02	3.39e + 02	4.93e + 02	2.97e + 02	**1.44e + 02**
	STD	1.53e + 02	1.76e + 02	1.47e + 02	2.60e + 02	1.12e + 02	**7.41e + 01**
F23	Mean	**3.15e + 02**	**3.15e + 02**	3.32e + 02	3.41e + 02	3.28e + 02	**3.15e + 02**
	STD	1.50e − 12	1.71e − 12	8.54e + 00	4.23e + 01	7.58e + 00	**5.05e − 13**
F24	Mean	**2.00e + 02**	**2.00e + 02**	**2.00e + 02**	2.38e + 02	**2.00e + 02**	2.31e + 02
	STD	6.37e − 04	**1.58e − 04**	8.09e − 04	5.42e + 01	7.42e − 04	5.73e + 00
F25	Mean	2.01e + 02	**2.00e + 02**	2.10e + 02	2.11e + 02	2.10e + 02	2.08e + 02
	STD	2.55e + 00	**0.00e + 00**	4.03e + 00	7.30e + 00	3.62e + 00	3.42e + 00
F26	Mean	1.30e + 02	1.25e + 02	1.30e + 02	1.44e + 02	1.34e + 02	**1.06e + 02**
	STD	4.59e + 01	4.22e + 01	4.58e + 01	4.96e + 01	4.74e + 01	**2.37e + 01**
F27	Mean	5.09e + 02	6.21e + 02	6.24e + 02	7.63e + 02	6.01e + 02	**4.51e + 02**
	STD	**6.92e + 01**	1.74e + 02	1.20e + 02	2.23e + 02	8.60e + 01	**7.28e + 01**
F28	Mean	9.71e + 02	1.14e + 03	1.05e + 03	1.38e + 03	9.22e + 02	**7.07e + 02**
	STD	1.43e + 02	2.69e + 02	2.46e + 02	6.95e + 02	1.08e + 02	**1.05e + 02**
F29	Mean	1.77e + 06	1.73e + 05	4.24e + 05	2.82e + 06	2.00e + 05	**5.29e + 02**
	STD	3.62e + 06	1.22e + 06	1.94e + 06	5.32e + 06	1.25e + 06	**1.62e + 02**
F30	Mean	3.15e + 03	3.00e + 03	3.99e + 04	2.91e + 04	2.19e + 04	**8.70e + 02**
	STD	9.36e + 02	1.13e + 03	2.88e + 04	4.56e + 04	1.27e + 04	**2.32e + 02**

**Table 3:** Results of the Wilcoxon test for the mean values in Table 2.

FSGWO v.s.	EO	MPSO	GWO	HPSOGWO	SOGWO
*p* value	8.3606e − 05	9.4199e − 06	2.7389e − 06	9.1269e − 07	3.3270e − 06

**Table 4:** Comparison of the calculation accuracy of related algorithms for 30 30D test functions.

FSGWO v.s.	EO	MPSO	GWO	HPSOGWO	SOGWO
F1	0.99233	0.99951	0.99993	0.99991	0.9999
F2	1	1	1	1	1
F3	1	1	1	1	1
F4	1	1	1	1	1
F5	0.01935	0.03823	0.04495	0.03886	0.04480
F6	−0.13328	0.35274	0.35262	0.43869	0.25254
F7	−0.46719	0.05726	0.99944	0.99886	0.99882
F8	1	1	1	1	1
F9	0.57994	0.46875	0.59616	0.61525	0.55491
F10	0.99135	0.98582	0.99384	0.99488	0.99289
F11	0.39112	0.32372	0.27480	0.45486	0.25834
F12	0.79911	0.61698	0.88735	0.86818	0.91712
F13	−0.26746	0.43291	0.28450	0.37861	0.16395
F14	0.17936	0.53175	0.90744	0.93668	0.77010
F15	−0.09313	0.32768	0.90905	0.99968	0.83011
F16	0.07097	0.14846	0.06430	0.12162	0.04669
F17	0.98757	0.89615	0.99838	0.99748	0.99735
F18	0.97451	0.86556	0.99998	0.99995	0.99998
F19	0.56795	0.51507	0.89798	0.91101	0.80831
F20	0.83864	0.79354	0.99622	0.99604	0.99456
F21	0.99531	0.98043	0.99946	0.99954	0.99864
F22	0.56651	0.65413	0.57670	0.70879	0.51583
F23	0	0	0.05049	0.07450	0.03775
F24	−0.15584	−0.15584	−0.15584	0.02768	−0.15584
F25	−0.03369	−0.03851	0.01218	0.01622	0.00905
F26	0.18118	0.14955	0.18180	0.26123	0.20560
F27	0.11363	0.27257	0.27601	0.40817	0.24936
F28	0.27247	0.38170	0.32701	0.48875	0.23333
F29	0.99970	0.99695	0.99875	0.99981	0.99735
F30	0.72346	0.70966	0.97818	0.97013	0.96032
Average	0.4698	0.5435	0.6484	0.6902	0.6227

**Table 5:** Results of the related algorithms for 50-dimensional functions.

Function	Index	EO	MPSO	GWO	HPSOGWO	SOGWO	FSGWO
F1	Mean	1.32e + 06	1.80e + 07	8.42e + 07	4.34e + 07	6.77e + 07	**2.66e + 04**
	STD	5.23e + 05	1.58e + 07	5.08e + 07	5.51e + 07	3.84e + 07	**1.75e + 04**
F2	Mean	7.35e + 03	1.72e + 09	7.67e + 09	5.63e + 09	4.14e + 09	**4.16e − 05**
	STD	8.57e + 03	1.68e + 09	3.29e + 09	1.12e + 10	3.21e + 09	**1.56e − 04**
F3	Mean	8.80e + 02	6.43e + 03	5.82e + 04	4.92e + 04	4.72e + 04	**1.14e − 02**
	STD	6.76e + 02	5.41e + 03	1.08e + 04	3.19e + 04	1.06e + 04	**3.64e − 02**
F4	Mean	8.15e + 01	2.11e + 02	7.64e + 02	5.68e + 02	4.50e + 02	**8.76e + 00**
	STD	3.63e + 01	2.67e + 02	3.33e + 02	8.90e + 02	2.02e + 02	**1.80e + 01**
F5	Mean	2.05e + 01	2.11e + 01	2.11e + 01	2.10e + 01	2.11e + 01	**2.00e + 01**
	STD	1.19e − 01	5.14e − 02	4.44e − 02	2.90e − 01	4.43e − 02	**8.68e − 03**
F6	Mean	**2.06e + 01**	3.00e + 01	2.98e + 01	2.81e + 01	2.62e + 01	2.37e + 01
	STD	3.65e + 00	4.68e + 00	3.98e + 00	1.04e + 01	**3.62e + 00**	4.21e + 00
F7	Mean	7.04e − 03	8.68e − 03	7.85e + 01	5.64e + 01	4.17e + 01	**6.42e − 03**
	STD	1.01e − 02	1.13e − 02	3.44e + 01	1.37e + 02	3.30e + 01	**7.64e − 03**
F8	Mean	1.28e + 02	6.97e + 01	1.88e + 02	1.75e + 02	1.69e + 02	**0.00e + 00**
	STD	2.51e + 01	1.66e + 01	3.04e + 01	7.77e + 01	2.30e + 01	**0.00e + 00**
F9	Mean	1.63e + 02	1.50e + 02	2.02e + 02	2.62e + 02	1.82e + 02	**1.03e + 02**
	STD	3.64e + 01	3.13e + 01	3.13e + 01	1.54e + 02	4.81e + 01	**1.75e + 01**
F10	Mean	3.67e + 03	2.13e + 03	5.70e + 03	6.37e + 03	5.02e + 03	**2.74e + 01**
	STD	9.87e + 02	6.28e + 02	8.02e + 02	2.35e + 03	7.68e + 02	**6.31e + 00**
F11	Mean	6.66e + 03	5.88e + 03	5.70e + 03	6.46e + 03	5.27e + 03	**4.33e + 03**
	STD	8.95e + 02	9.05e + 02	1.37e + 03	2.68e + 03	1.32e + 03	**4.57e + 02**
F12	Mean	1.49e + 00	4.53e − 01	1.92e + 00	1.88e + 00	2.55e + 00	**1.86e − 01**
	STD	3.89e − 01	1.98e − 01	1.66e + 00	1.56e + 00	1.43e + 00	**3.26e − 02**
F13	Mean	**4.42e − 01**	5.85e − 01	7.46e − 01	8.03e − 01	5.92e − 01	4.69e − 01
	STD	8.05e − 02	2.92e − 01	5.09e − 01	8.17e − 01	**7.78e − 02**	8.04e − 02
F14	Mean	2.98e − 01	4.13e − 01	1.48e + 01	8.34e + 00	4.40e + 00	**2.88e − 01**
	STD	1.16e − 01	1.69e − 01	1.33e + 01	2.49e + 01	7.24e + 00	**7.33e − 02**
F15	Mean	**1.15e + 01**	2.57e + 01	1.65e + 03	4.39e + 03	5.33e + 02	2.44e + 01
	STD	**3.81e + 00**	8.01e + 00	2.36e + 03	2.31e + 04	9.96e + 02	6.05e + 00
F16	Mean	2.02e + 01	2.16e + 01	2.00e + 01	2.09e + 01	1.98e + 01	**1.88e + 01**
	STD	9.71e − 01	5.97e − 01	8.13e − 01	1.11e + 00	1.06e + 00	**5.18e − 01**
F17	Mean	2.58e + 05	3.75e + 05	4.55e + 06	1.97e + 06	3.00e + 06	**4.68e + 04**
	STD	1.40e + 05	8.94e + 05	5.24e + 06	2.10e + 06	1.83e + 06	**4.13e + 04**
F18	Mean	2.53e + 03	1.72e + 03	6.52e + 07	8.26e + 07	2.77e + 07	**4.84e + 02**
	STD	1.32e + 03	2.13e + 03	1.27e + 08	3.36e + 08	6.07e + 07	**5.70e + 02**
F19	Mean	**1.73e + 01**	2.56e + 01	8.25e + 01	7.04e + 01	7.35e + 01	2.57e + 01
	STD	**9.38e + 00**	1.47e + 01	2.84e + 01	4.70e + 01	2.36e + 01	2.05e + 01
F20	Mean	5.69e + 02	5.51e + 02	1.51e + 04	1.86e + 04	1.01e + 04	**2.76e + 02**
	STD	1.43e + 02	2.18e + 02	7.77e + 03	3.12e + 04	6.06e + 03	**3.02e + 02**
F21	Mean	1.76e + 05	1.79e + 05	2.62e + 06	1.77e + 06	2.18e + 06	**1.96e + 04**
	STD	1.10e + 05	2.00e + 05	2.76e + 06	3.37e + 06	1.85e + 06	**3.94e + 04**
F22	Mean	8.33e + 02	1.11e + 03	8.07e + 02	9.67e + 02	7.23e + 02	**4.59e + 02**
	STD	3.12e + 02	3.19e + 02	2.80e + 02	5.13e + 02	3.05e + 02	**1.68e + 02**
F23	Mean	3.45e + 02	3.45e + 02	4.37e + 02	3.94e + 02	4.15e + 02	**3.44e + 02**
	STD	1.01e − 03	1.09e − 12	4.22e + 01	6.97e + 01	3.00e + 01	**8.44e − 13**
F24	Mean	2.01e + 02	2.20e + 02	2.01e + 02	2.63e + 02	**2.00e + 02**	2.86e + 02
	STD	5.47e − 04	3.13e + 01	6.47e − 04	8.22e + 01	**4.88e − 04**	5.50e + 00
F25	Mean	**2.00e + 02**	2.01e + 02	2.27e + 02	2.27e + 02	2.23e + 02	2.28e + 02
	STD	**2.90e − 13**	4.06e + 00	9.21e + 00	1.61e + 01	6.45e + 00	7.02e + 00
F26	Mean	1.80e + 02	1.91e + 02	1.88e + 02	1.94e + 02	1.80e + 02	**1.04e + 02**
	STD	4.00e + 01	2.94e + 01	4.43e + 01	8.37e + 01	4.98e + 01	**1.95e + 01**
F27	Mean	**8.55e + 02**	1.20e + 03	1.06e + 03	1.17e + 03	9.35e + 02	9.67e + 02
	STD	**9.36e + 01**	1.51e + 02	1.11e + 02	3.21e + 02	1.10e + 02	8.51e + 01
F28	Mean	1.57e + 03	2.32e + 03	2.18e + 03	2.51e + 03	1.88e + 03	**1.53e + 03**
	STD	3.56e + 02	7.20e + 02	5.22e + 02	1.71e + 03	4.86e + 02	**2.05e + 02**
F29	Mean	1.71e + 07	2.25e + 06	4.98e + 06	2.12e + 07	1.07e + 06	**9.36e + 02**
	STD	2.08e + 07	1.17e + 07	7.89e + 06	3.84e + 07	2.45e + 06	**1.17e + 02**
F30	Mean	1.12e + 04	2.25e + 04	1.44e + 05	6.35e + 04	1.01e + 05	**1.09e + 04**
	STD	1.89e + 03	1.44e + 04	9.16e + 04	8.40e + 04	4.86e + 04	**1.06e + 03**

**Table 6:** Results of the Wilcoxon test for the mean values of Table 5.

FSGWO v.s.	EO	MPSO	GWO	HPSOGWO	SOGWO
*p* value	6.2062e−04	1.3587e − 05	4.0348e − 06	2.7389e − 06	1.4875e − 05

**Table 7:** Comparison of the calculation accuracy of related algorithms for 30 50-D test functions.

FSGWO v.s.	EO	MPSO	GWO	HPSOGWO	SOGWO
F1	0.97980	0.99851	0.99968	0.99938	0.99960
F2	0.99999	0.99999	0.99999	0.99999	0.99999
F3	0.99998	0.99999	0.99999	0.99999	0.99999
F4	0.89254	0.95845	0.98853	0.98457	0.98052
F5	0.02433	0.04952	0.05230	0.04873	0.05284
F6	−0.15302	0.21058	0.20577	0.15643	0.09618
F7	0.08817	0.25980	0.99991	0.99988	0.99984
F8	1	1	1	1	1
F9	0.36540	0.31038	0.48843	0.60660	0.43276
F10	0.99251	0.98709	0.99518	0.99569	0.99453
F11	0.34982	0.26415	0.24015	0.32972	0.17841
F12	0.87485	0.58948	0.90300	0.90099	0.92698
F13	−0.06079	0.19856	0.37149	0.41602	0.20706
F14	0.03271	0.30191	0.98054	0.96542	0.93453
F15	−1.12418	0.05005	0.98515	0.99442	0.95411
F16	0.06779	0.12730	0.05831	0.09673	0.04623
F17	0.81835	0.87511	0.98971	0.97621	0.98438
F18	0.80887	0.71945	0.99999	0.99999	0.99998
F19	−0.48750	−0.00178	0.68877	0.63547	0.65086
F20	0.51465	0.49930	0.98170	0.98517	0.97274
F21	0.88885	0.89059	0.99250	0.98892	0.99102
F22	0.44841	0.58529	0.43070	0.525	0.36459
F23	0	0	0.21257	0.12794	0.17198
F24	−0.42928	−0.30017	−0.42928	−0.08550	−0.42928
F25	−0.14177	−0.13853	−0.00643	−0.00486	−0.02340
F26	0.42134	0.45188	0.44399	0.46109	0.41925
F27	−0.13037	0.19254	0.08732	0.17541	−0.03359
F28	0.02665	0.34062	0.29742	0.38918	0.18338
F29	0.99994	0.99958	0.99981	0.99995	0.99912
F30	0.02232	0.51392	0.92418	0.82759	0.89163
Average	0.3363	0.4645	0.6294	0.6499	0.5982

**Table 8:** Results of FSGWO and FSGWO1 for 30-dimensional functions.

Function	Index	FSGWO	FSGWO1	Function	Index	FSGWO	FSGWO1
F1	Mean	**3.29e + 03**	5.74e + 04	F16	Mean	**1.03e + 01**	1.07e + 01
	STD	**3.46e + 03**	1.06e + 05		STD	**3.73e − 01**	3.98e − 01
F2	Mean	**0.00e + 00**	**0.00e + 00**	F17	Mean	2.32e + 03	**1.97e + 03**
	STD	**0.00e + 00**	**0.00e + 00**		STD	4.37e + 03	**1.10e + 03**
F3	Mean	**0.00e + 00**	**0.00e + 00**	F18	Mean	**7.26e + 01**	8.39e + 01
	STD	**0.00e + 00**	**0.00e + 00**		STD	3.08e + 01	**2.74e + 01**
F4	Mean	**0.00e + 00**	2.86e + 01	F19	Mean	**3.95e + 00**	8.36e + 00
	STD	**0.00e + 00**	3.58e + 01		STD	**1.13e + 00**	8.44e + 00
F5	Mean	**2.00e + 01**	2.05e + 01	F20	Mean	5.73e + 01	**4.86e + 01**
	STD	**3.28e − 03**	5.22e − 02		STD	3.14e + 01	**2.26e + 01**
F6	Mean	8.33e + 00	**4.23e + 00**	F21	Mean	**4.10e + 02**	6.00e + 02
	STD	3.57e + 00	**1.38e + 00**		STD	**2.49e + 02**	5.80e + 02
F7	Mean	**7.86e − 03**	1.35e − 02	F22	Mean	**1.44e + 02**	1.78e + 02
	STD	**1.21e − 02**	1.37e − 02		STD	7.41e + 01	**6.66e + 01**
F8	Mean	**0.00e + 00**	1.48e + 01	F23	Mean	**3.15e + 02**	**3.15e + 02**
	STD	**0.00e + 00**	2.83e + 00		STD	**5.05e − 13**	5.94e − 13
F9	Mean	**3.71e + 01**	4.94e + 01	F24	Mean	2.31e + 02	**2.29e + 02**
	STD	8.27e + 00	**7.51e + 00**		STD	5.73e + 00	**5.55e + 00**
F10	Mean	**1.35e + 01**	3.50e + 02	F25	Mean	**2.08e + 02**	2.09e + 02
	STD	**3.73e + 00**	1.01e + 02		STD	3.42e + 00	**2.83e + 00**
F11	Mean	**1.98e + 03**	3.00e + 03	F26	Mean	**1.06e + 02**	1.24e + 02
	STD	3.09e + 02	**2.77e + 02**		STD	**2.37e + 01**	4.27e + 01
F12	Mean	**1.84e − 01**	5.66e − 01	F27	Mean	4.51e + 02	**4.28e + 02**
	STD	**3.67e − 02**	8.91e − 02		STD	7.28e + 01	**5.05e + 01**
F13	Mean	2.78e − 01	**2.64e − 01**	F28	Mean	**7.07e + 02**	8.76e + 02
	STD	6.68e − 02	**5.31e − 02**		STD	1.05e + 02	**4.81e + 01**
F14	Mean	**2.10e − 01**	2.29e − 01	F29	Mean	**5.29e + 02**	7.69e + 02
	STD	4.68e − 02	**4.60e − 02**		STD	1.62e + 02	**1.39e + 02**
F15	Mean	**4.89e + 00**	7.18e + 00	F30	Mean	**8.70e + 02**	1.99e + 03
	STD	**1.31e + 00**	1.49e + 00		STD	**2.32e + 02**	6.60e + 02

**Table 9:** Results of the Wilcoxon test for the mean values of Table 8.

FSGWO v.s.	FSGWO1
*p* value	2.7610e − 03

**Table 10:** Results of FSGWO and FSGWO2 for 30-dimensional functions.

Function	Index	FSGWO	FSGWO2	Function	Index	FSGWO	FSGWO2
F1	Mean	**3.29e + 03**	1.09e + 04	F16	Mean	1.03e + 01	**1.02e + 01**
	STD	**3.46e + 03**	7.88e + 03		STD	3.73e − 01	**3.38e − 01**
F2	Mean	**0.00e + 00**	**0.00e + 00**	F17	Mean	**2.32e + 03**	5.21e + 03
	STD	**0.00e + 00**	**0.00e + 00**		STD	4.37e + 03	8.12e + 03
F3	Mean	**0.00e + 00**	**0.00e + 00**	F18	Mean	**7.26e + 01**	7.55e + 01
	STD	**0.00e + 00**	**0.00e + 00**		STD	3.08e + 01	**2.73e + 01**
F4	Mean	**0.00e + 00**	1.37e + 00	F19	Mean	**3.95e + 00**	8.66e + 00
	STD	**0.00e + 00**	9.10e + 00		STD	**1.13e + 00**	1.18e + 01
F5	Mean	**2.00e + 01**	2.02e + 01	F20	Mean	5.73e + 01	**4.03e + 01**
	STD	**3.28e−03**	3.82e − 02		STD	3.14e + 01	**2.64e + 01**
F6	Mean	8.33e + 00	**3.28e + 00**	F21	Mean	**4.10e + 02**	5.49e + 02
	STD	3.57e + 00	**2.56e + 00**		STD	**2.49e + 02**	7.45e + 02
F7	Mean	**7.86e − 03**	1.08e − 02	F22	Mean	**1.44e + 02**	1.67e + 02
	STD	**1.21e − 02**	1.41e − 02		STD	7.41e + 01	**6.17e + 01**
F8	Mean	**0.00e + 00**	7.08e − 02	F23	Mean	**3.15e + 02**	**3.15e + 02**
	STD	**0.00e + 00**	5.05e − 01		STD	**5.05e − 13**	5.35e − 13
F9	Mean	**3.71e + 01**	4.48e + 01	F24	Mean	**2.31e + 02**	**2.31e + 02**
	STD	8.27e + 00	**7.67e + 00**		STD	**5.73e + 00**	6.83e + 00
F10	Mean	**1.35e + 01**	9.25e + 01	F25	Mean	2.08e + 02	**2.07e + 02**
	STD	**3.73e + 00**	3.84e + 01		STD	3.42e + 00	**2.78e + 00**
F11	Mean	**1.98e + 03**	2.63e + 03	F26	Mean	**1.06e + 02**	1.20e + 02
	STD	3.09e + 02	**2.28e + 02**		STD	**2.37e + 01**	4.00e + 01
F12	Mean	**1.84e − 01**	3.75e − 01	F27	Mean	4.51e + 02	**4.25e + 02**
	STD	**3.67e − 02**	5.87e − 02		STD	7.28e + 01	**4.79e + 01**
F13	Mean	2.78e − 01	**2.46e − 01**	F28	Mean	**7.07e + 02**	8.74e + 02
	STD	6.68e − 02	**5.89e − 02**		STD	1.05e + 02	**5.15e + 01**
F14	Mean	**2.10e − 01**	2.13e − 01	F29	Mean	**5.29e + 02**	6.58e + 02
	STD	**4.68e − 02**	5.06e − 02		STD	**1.62e + 02**	1.66e + 02
F15	Mean	**4.89e + 00**	6.04e + 00	F30	Mean	**8.70e + 02**	1.69e + 03
	STD	1.31e + 00	**1.26e + 00**		STD	**2.32e + 02**	5.83e + 02

**Table 11:** Results of the Wilcoxon test for the mean values of Table 10.

FSGWO v.s.	FSGWO1
*p* value	2.9719e − 03

**Table 12:** Results of the related algorithms for the 40-unit ELD case.

No.	Algorithm	Best	Mean	Median	Worst	STD
1	FSGWO	**1.2260e + 05**	**1.2514e + 05**	**1.2519e + 05**	1.2758e + 05	9.8099e + 02
2	GWO	1.2602e + 05	1.2762e + 05	1.2751e + 05	1.3002e + 05	9.4627e + 02
3	HPSOGWO	1.2474e + 05	1.2614e + 05	1.2606e + 05	1.2795e + 05	1.0180e + 03
4	SOGWO	1.2626e + 05	1.2804e + 05	1.2799e + 05	1.3113e + 05	1.3027e + 03
5	EO	1.2591e + 05	1.2725e + 05	1.2688e + 05	1.2930e + 05	1.0265e + 03
6	MPSO	1.2617e + 05	1.2741e + 05	1.2737e + 05	1.2920e + 05	**7.4119e + 02**
7	iHS [52]	1.2980e + 05	1.3375e + 05	1.3392e + 05	1.3701e + 05	1.6258e + 03
8	IMO [53]	1.3073e + 05	1.3465e + 05	1.3428e + 05	1.3846e + 05	2.2328e + 03
9	MIMO [53]	1.2960e + 05	1.3306e + 05	1.3297e + 05	1.3698e + 05	2.1161e + 03
10	APS 9 [54]	1.2390e + 05	1.2553e + 05	1.2544e + 05	**1.2715e + 05**	8.0868e + 02
11	ESSA [55]	1.2885e + 05	1.3061e + 05	—	1.3355e + 05	1.0434e + 03
12	GA-MPC [56]	1.2921e + 05	1.3323e + 05	1.3319e + 05	1.3606e + 05	1.8788e + 03

**Table 13:** Results of the related algorithms for the 140-unit ELD case.

No.	Algorithm	Best	Mean	Median	Worst	STD
1	FSGWO	**1.7551e + 06**	**1.8119e + 06**	**1.8107e + 06**	**1.8598e + 06**	2.3481e + 04
2	GWO	1.8935e + 06	1.9313e + 06	1.9317e + 06	1.9582e + 06	1.6986e + 04
3	HPSOGWO	1.8611e + 06	1.9210e + 06	1.9234e + 06	1.9728e + 06	3.0071e + 04
4	SOGWO	1.8828e + 06	1.9303e + 06	1.9284e + 06	1.9660e + 06	2.0406e + 04
5	EO	1.8645e + 06	1.9187e + 06	1.9190e + 06	1.9639e + 06	2.3944e + 04
6	MPSO	1.8756e + 06	1.9173e + 06	1.9133e + 06	1.9746e + 06	2.4482e + 04
7	iHS [52]	1.9142e + 06	2.0538e + 06	1.9942e + 06	2.5494e + 06	1.5139e + 05
8	IMO [53]	1.9061e + 06	1.9338e + 06	1.9322e + 06	1.9639e + 06	1.6741e + 04
9	MIMO [53]	1.8957e + 06	1.9181e + 06	1.9184e + 06	1.9373e + 06	**1.1350e + 04**
10	APS 9 [54]	1.8540e + 06	2.0666e + 06	1.9268e + 06	2.9513e + 06	3.1309e + 05
11	ESSA [55]	1.9087e + 06	1.9350e + 06	—	1.9541e + 06	1.3123e + 04
12	GA-MPC [56]	1.9203e + 06	1.9533e + 06	1.9567e + 06	1.9707e + 06	1.4084e + 04

**Table 14:** Results of related algorithms for the three-bar truss design problem.

Algorithm	*x*_1_	*x*_2_	*f* (*x*_1,_*x*_2_)
FSGWO	0.7886751	0.4082485	**263.8958**
m-GWO [58]	0.7885845	0.4085071	263.8961
m-SCA [59]	0.81915	0.36956	263.8972
MFO [60]	0.78824477	0.40946691	263.8960
CS [57]	0.78867	0.40902	263.9716

**Table 15:** Results of related algorithms for the pressure vessel design problem.

Algorithm	*x*_1_	*x*_2_	*x*_3_	*x*_4_	*f*(*x*_1_,*x*_2_,*x*_3_,*x*_4_)
FSGWO	0.7782	0.3846	40.3196	200.0000	**5885.3328**
BGWO [61]	0.7783	0.3847	40.3197	200.0000	5886.4955
I-GWO [62]	0.779031	0.385501	40.36313	199.4017	5888.3400
BFGSOLMFO [63]	0.778675	0.385392	40.342876	199.754805	5889.7080
SMA [64]	0.7931	0.3932	40.6711	196.2178	5994.1857

**Table 16:** Results of related algorithms for the gear train design problem.

Algorithm	*x*_1_	*x*_2_	*x*_3_	*x*_4_	*f*(*x*_1_,*x*_2_,*x*_3_,*x*_4_)
FSGWO	19	43	16	49	**2.7009e−12**
m-SCA [59]	43	16	19	49	**2.7009e−12**
CS [57]	43	16	19	49	**2.7009e−12**
LPE [65]	19	49	16	43	**2.7009e−12**
GWOSCA [66]	26	51	15	53	2.3078e−11

**Table 17:** Results of related algorithms for the cantilever beam design problem.

Algorithm	*x*_1_	*x*_2_	*x*_3_	*x*_4_	*x*_5_	*f*(*x*_1_,*x*_2_,*x*_3_,*x*_4_,*x*_5_)
FSGWO	6.0160	5.3092	4.4943	3.5015	2.1527	**1.33996**
CS [57]	6.0089	5.3049	4.5023	3.5077	2.1504	1.33999
BGWO [61]	6.0130	5.3112	4.4953	3.5079	2.1461	**1.33996**
m-SCA [59]	6.0089	5.3049	4.5023	3.5077	2.1504	1.33999
MFO [60]	5.9849	5.3167	4.4973	3.5136	2.1616	1.33999

## Data Availability

Not applicable.
